# Perspectives on Advanced Lithium–Sulfur Batteries for Electric Vehicles and Grid-Scale Energy Storage

**DOI:** 10.3390/nano14120990

**Published:** 2024-06-07

**Authors:** Wei Ni

**Affiliations:** State Key Laboratory of Vanadium and Titanium Resources Comprehensive Utilization, ANSTEEL Research Institute of Vanadium & Titanium (Iron & Steel), Chengdu 610031, China; niwei@iccas.ac.cn or max.ni@hotmail.com

**Keywords:** lithium–sulfur batteries, Li–S batteries, power batteries, electric vehicles, grid energy storage, large-scale energy storage, renewable energy integration

## Abstract

Intensive increases in electrical energy storage are being driven by electric vehicles (EVs), smart grids, intermittent renewable energy, and decarbonization of the energy economy. Advanced lithium–sulfur batteries (LSBs) are among the most promising candidates, especially for EVs and grid-scale energy storage applications. In this topical review, the recent progress and perspectives of practical LSBs are reviewed and discussed; the challenges and solutions for these LSBs are analyzed and proposed for future practical and large-scale energy storage applications. Major challenges for the shuttle effect, reaction kinetics, and anodes are specifically addressed, and solutions are provided on the basis of recent progress in electrodes, electrolytes, binders, interlayers, conductivity, electrocatalysis, artificial SEI layers, etc. The characterization strategies (including in situ ones) and practical parameters (e.g., cost-effectiveness, battery management/modeling, environmental adaptability) are assessed for crucial automotive/stationary large-scale energy storage applications (i.e., EVs and grid energy storage). This topical review will give insights into the future development of promising Li–S batteries toward practical applications, including EVs and grid storage.

## 1. Introduction

**Figure 1:**
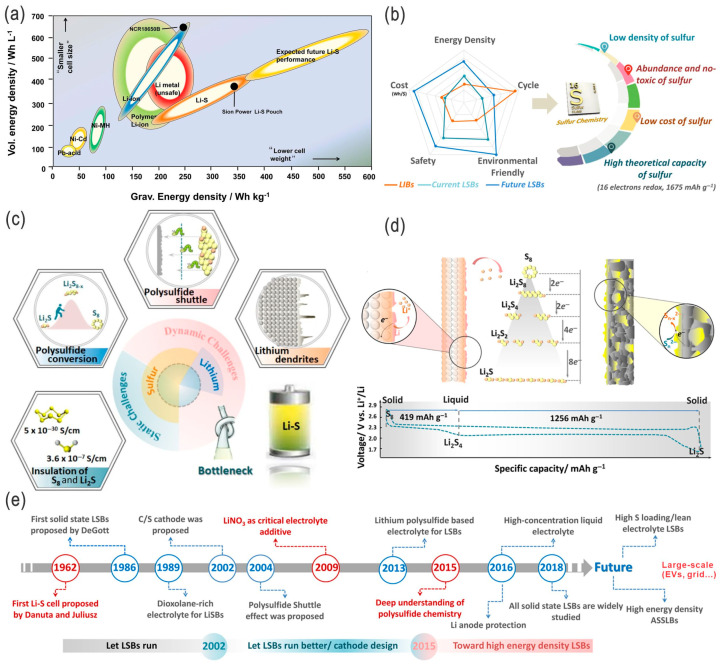
(**a**) Gravimetric and volumetric energy densities of various electrochemical energy storage systems. Adapted with permission from Ref. [11]. Copyright 2015 WILEY-VCH Verlag GmbH & Co. KGaA, Weinheim. (**b**) Radar diagram of the current and near-future LSBs (compared to LIBs), as well as the advantages of LSBs. (**c**) Static and dynamic challenges toward practical LSBs. (**d**) Redox reaction in LSBs and corresponding charging–discharging profile, and (**e**) main milestones of development in LSB research. Adapted with permission from Ref. [4]. Copyright 2022 the authors. Published by UESTC and John Wiley & Sons Australia, Ltd. Open access under the terms of the Creative Commons Attribution License.

**Figure 2:**
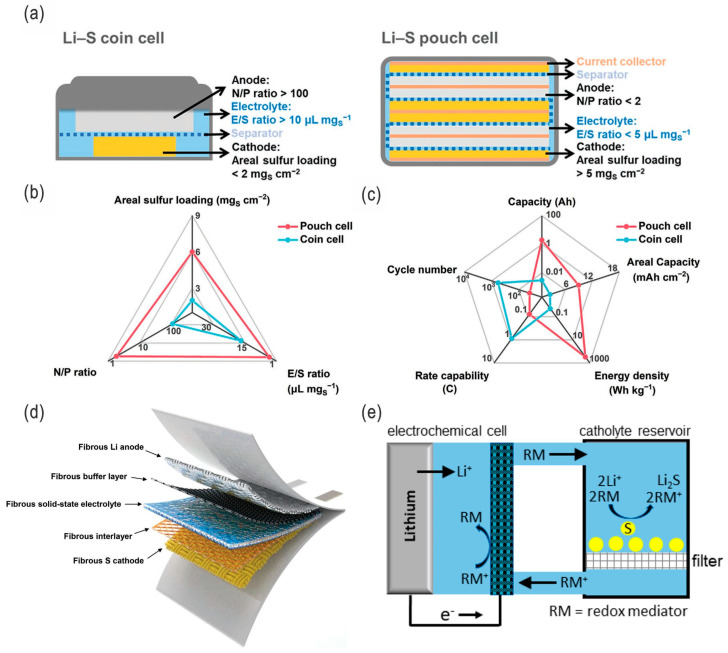
(**a**–**c**) Comparison between Li–S coin cells and pouch cells: schematic illustration, design parameters, and cell performances, respectively. Adapted with permission from Ref. [12]. Copyright 2022 Wiley-VCH GmbH. (**d**) Schematic illustration of a flexible Li–S cell fabricated with fibrous materials, which concurrently serve as flexible current collectors of the S cathode and Li anode, as well as interfacial layers (e.g., interlayers, buffer layers, and solid-state electrolytes). Adapted with permission from Ref. [13]. Copyright 2020 Wiley-VCH GmbH. (**e**) Schematic illustration of a Li–S flow cell for potential grid-scale energy storage. Adapted with permission from Ref. [14]. Copyright 2022 the authors. Published by the American Chemical Society. Open access licensed under CC-BY-NC-ND 4.0.

Due to the increasing interest in clean energy storage and conversion, as well as in decarbonizing the energy economy, effective, low-cost, high-performance, and scalable electrical energy storage technologies, materials, and systems are favorable and highly desirable [1]. Compared with Li-ion batteries (LIBs), Li–S batteries (LSBs) have distinct advantages and also present unique challenges. These major advantages include unprecedented energy density (2600 Wh kg^−1^ and 2800 Wh L^−1^ for sulfur cathodes [2]), lightweight efficiency, lower cost, environmental friendliness, and safety, as well as comparatively quick charging capability, cycle life, coulombic efficiency (CE), and self-discharge with the significant progress in recent years, thus setting the stage for a new revolution in the world of electric vehicles and grid energy storage (the main features, advantages/challenges, and development milestones of LSBs are schematically presented in Figure 1) [1,3,4,5,6,7,8]. In addition to their great potential commercial applications in EVs, LSBs are also among the most promising electrical energy storage systems for large-scale grid storage, which could more easily regulate supply and demand by decoupling electricity generation and loads, as well as improving the grid security by distributed storage. Although the voltage output of LSBs is around 2.2 V (about 60% of the 3.7 V for conventional LIBs, usually lower than the 4.3 V stability windows of electrolytes), their significantly high theoretical energy density is 2600 Wh kg^−1^ vs. 600 Wh kg^−1^ for the current LIBs, based on the high specific capacity of sulfur (1675 mAh g^−1^ vs. 130–200 mAh g^−1^ for the current cathode materials of LIBs), which makes it 4–5 times higher than for the conventional LIBs system, thus potentially providing a driving range of nearly 300 miles (or 480 km) and an alternative form of stationary energy storage [1,9]. There are several types of LSBs, mainly including coin cell, pouch cell [10], flexible, and flow-type (Figure 2), although the latter three may be more promising for large-scale energy storage.

## 2. Major Challenges and Solutions

The major and critical challenges encountered by LSBs for their wide practical application result from the poor electronic conductivity of sulfur and the shuttling effects of various soluble polysulfides, Li_2_S*_x_* (4 ≤ *x* ≤ 8, which gradually resolve in the electrolyte and transfer through the separator to the anode/negative electrode side, suffering a possible parasitic reaction with Li; during the charging process, the passivation layers, i.e., the low-conductivity Li_2_S/Li_2_S_2_, may deposit on the cathode/positive electrode), which lowers the utilization ratio (or even permanent capacity fade), self-discharge/short life cycle, cell impedance, and coulombic efficiency of LSBs [1,15,16]. Also, the high volume change (up to 76%) during the multiple conversion reaction of the sulfur cathode can deteriorate the integration of the electrode and result in capacity loss upon repeated charge–discharge processes [1]. Furthermore, the dendrite issue on the Li metal anode is another obstacle encountered in the practical application of LSBs [1,10]. Other challenges include reducing the amount of electrolyte while maintaining stable performance, i.e., using lean electrolytes (also called sparingly solvating electrolytes) rather than flooded/excessive electrolytes to enhance the specific energy and reduce the cost of the LSBs [17].

**Figure 3:**
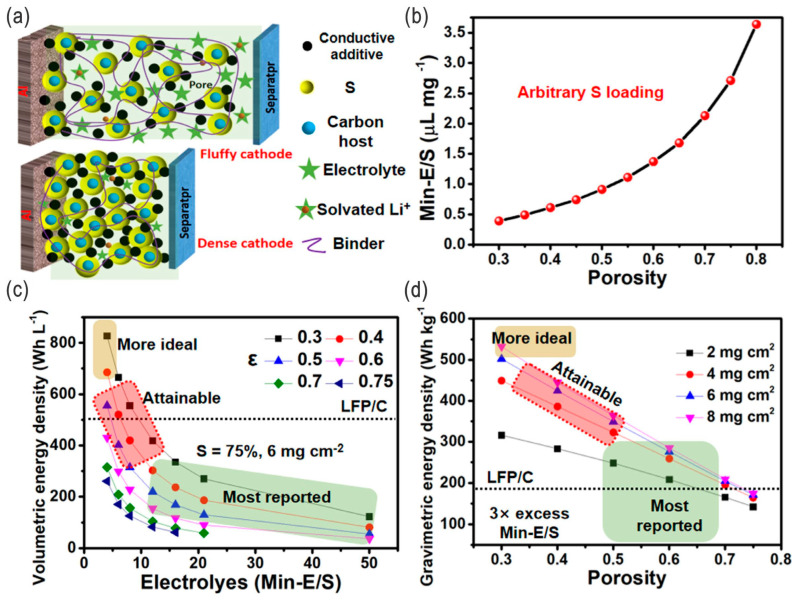
(**a**) Schematic illustration of the fluffy and dense cathodes. (**b**) The effect of cathode porosity on the minimum E/S ratio. (**c**,**d**) The predicted cell-level volumetric energy density (*E*_v_) and gravimetric energy density (*E*_g_) of LSBs, respectively, which are affected by cathode porosity, E/S ratio, sulfur loading, etc. Adapted with permission from Ref. [24]. Copyright 2021 Elsevier B.V.

To date, many different strategies have been proposed to address these obstacles for next-generation high-performance practical LSBs [1,18], e.g., nanostructuring of materials (nanoengineering) [19,20,21,22], compositing/hybridization, modification of cathode materials and Li metal anodes (including interface engineering [23]), introduction of interlayers and electrocatalysts, and optimization of electrolytes. For example, the energy densities of LSBs could be significantly enhanced by synergistic electrode/electrolyte engineering (Figure 3) [24].

### 2.1. Shuttle Effect

The shuttle effect is the main obstacle toward practical applications and may be suppressed or even eliminated by different strategies, including cathode host construction/modification [25], interlayers, separator modification, electrolyte optimization, and novel binders [26].

#### 2.1.1. Electrodes

The conventional sulfur powder has once again been intensively investigated since 2009 (i.e., Nazar’s group’s work on mesoporous carbon/PEG composite framework-hosted sublimed sulfur [27]), experiencing a renaissance and having made great progress toward practical applications [3]. Carbon materials (including porous carbon, carbon nanotubes, and graphene, with additional regulatory roles in the lithium storage behavior of electroactive materials and/or the construction of crack-free electrodes, and increasing the range of applications of LSBs [28,29]), as well as conductive polymers, are traditional hosts for sulfur cathode materials. In addition to the conductive materials, some polar and polysulfide-philic semi-conductive materials, including transition-metal oxides/chalcogenides (e.g., mesoporous TiO_2_), can also serve as advanced host materials for sulfur, although some conductive components may be added in the composite or cathode for better electric conductivity.

Chemical modification of cathode matrices with polysulfide-philic molecules/chemical groups can enhance the absorption of polysulfide active materials and further restrain the sulfur loss from the cathode. CTAB-modified graphene oxides (CTAB: cetyltrimethylammonium bromide) [30] and amino-functionalized rGO (by adding ethylenediamine (EDA); rGO: reduced graphene oxide) [31] are among these typical and effective examples. These optimized conductive cathode matrices’ strong binding to soluble Li_2_S*_x_* (*x* > 2) enables higher capacity retention over long cycles, with high capacities together with high (rate) capabilities. Although chemical stabilization of sulfur cathodes is another way to bond the sulfur species and, thus, achieve a more stable cycling performance even in a lean electrolyte, these sulfur molecules bond tightly by forming strong chemical bonds between sulfur and oxygen/carbon (i.e., O–S and C–S) [32], e.g., in an heteroatom-rich carbon host, somewhat lowering the voltage output and the energy densities thereof.

**Figure 4:**
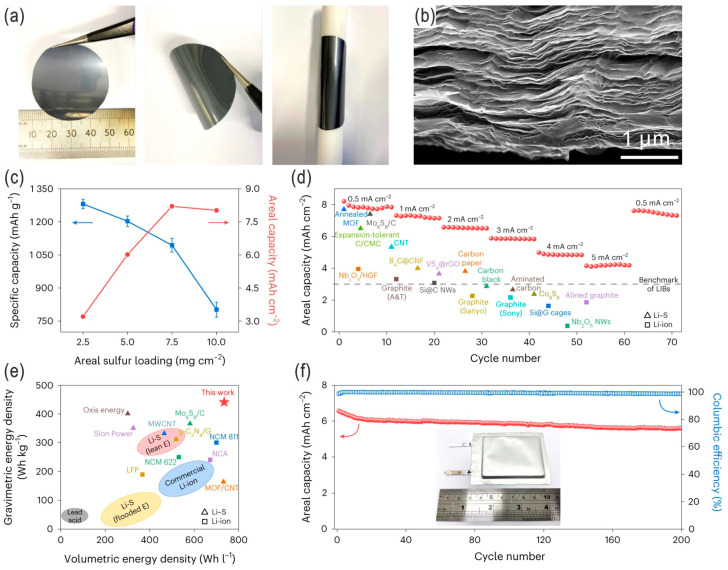
(**a**,**b**) Photos and SEM image, respectively, of a freestanding and flexible Li*_x_*MoS_2_ film consisting of laminar stacked nanosheets. Performances of Li*_x_*MoS_2_-based Li–S pouch cells: (**c**) Specific capacity retention and areal capacities with different sulfur loading in the Li*_x_*MoS_2_ cathode host (optimal loading: 7.5 mg cm^−2^). (**d**) Rate performances of reported Li–S batteries and LIBs using different types of materials. (**e**) Comparison of the gravimetric and volumetric energy densities of Li*_x_*MoS_2_-based Ah-level pouch cells with reported Li–S batteries, commercial LIBs, and lead–acid batteries (calculated on the basis of entire device configuration). (**f**) Cycling performance of the as-fabricated Li*_x_*MoS_2_-based Li–S pouch cell (inset: prototype photo) at 2 mA cm^−2^. Adapted with permission from Ref. [33]. Copyright 2023 the author(s), under exclusive license to Springer Nature Limited.

Some newer conductive materials, such as 2D conductive MXenes and MoS_2_, are also emerging in recent years. For example, Li et al. fabricated a kind of lithiated metallic molybdenum disulfide (2D Li*_x_*MoS_2_) nanosheets as a sulfur host material by prelithiation of metallic 1T-phase 2D MoS_2_ (Figure 4a,b) [33]. The 2D Li*_x_*MoS_2_ nanosheet-based Li–S batteries under a lean electrolyte showed high performances, including high energy density of 441 Wh kg^−1^ and 735 Wh L^−1^, as well as a capacity of 85.2% over 200 cycles with high sulfur loading in a pouch cell (Figure 4c,d). The high energy densities can be attributed to the enhanced adsorption of lithium polysulfides (LPSs), accelerating Li^+^ transport together with the electrocatalytic activity and reaction kinetics of LPSs by the replacement of the conductive and lyophilic 2D materials of prelithiated MoS_2_ as a bifunctional sulfur cathode host. Loading of sulfur-based active materials on (porous) carbon cloth/paper or conductive scaffolds (e.g., carbon nanotube paper, graphene films, carbon nanofiber sheets) will eliminate the use of conductive agents (e.g., carbon black, Ketjen black, active carbon, carbon nanotubes), binders, and even current collectors [28,34,35,36,37], which thus possess higher energy densities and are simultaneously versatile for the design of flexible Li–S batteries [13], in addition to the conventional pouch cells. However, the electrolyte optimization and electrode nanoengineering/modification are required for practically promising LSBs with higher rate capabilities and energy density [36,38].

Small sulfur molecules have promised better Li–S batteries, and these metastable S_2–4_ sulfur molecules [39,40] or intermediates [41] confined in the micropores of carbon substrates are one of the effective strategies to combat sulfur loss as well as poor electronic conductivity of sulfur cathodes, even in traditional carbonate electrolytes (e.g., EC/DEC). However, the small sulfur molecules or microspores usually result in slightly sacrificed voltage output (~0.2 V), although the long-term cycling stability is greatly enhanced.

**Figure 5:**
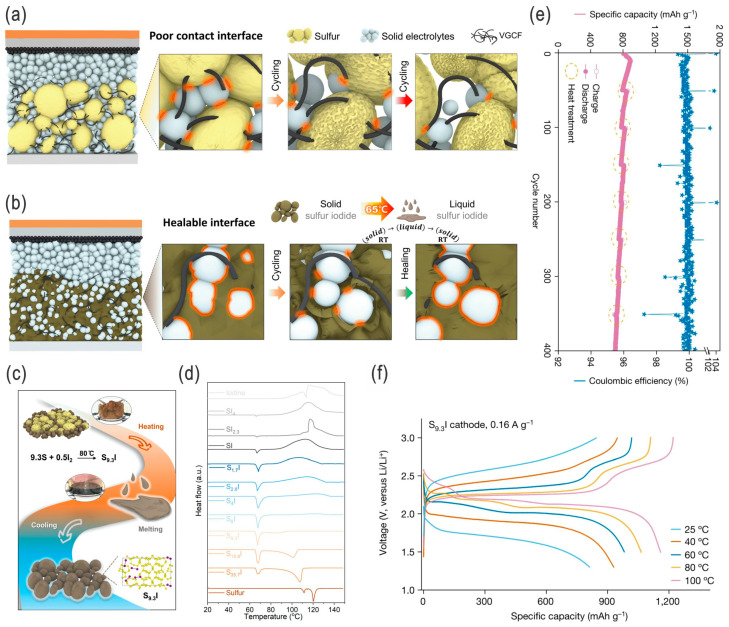
(**a**) Healable interfaces in solid-state Li–S batteries (SSLSBs) with a low-melting-point sulfur iodide, and the synthesis and characterization of sulfur iodide materials: (**a**,**b**) Schematic illustrations of SSLSBs with elemental sulfur and sulfur iodide as the active materials, respectively, exhibiting corresponding poor solid–solid contact and ideal healable active material–electrolyte interfaces. (**c**,**d**) Schematic illustration of the synthesis of S_9.3_I, and the DSC curves of sulfur iodide with varying S/I ratios, respectively. (**e**) Long-term cycling performance of S_9.3_I cathode at 0.16 A g^−1^ (25 °C; asterisks denote abnormal CE values corresponding to periodical repair by heat treatment), and (**f**) discharge–charge profiles of S_9.3_I cathode at 0.16 A g^−1^ from 25 to 100 °C in SSLSBs. Adapted with permission from Ref. [43]. Copyright 2024 the author(s), under exclusive license to Springer Nature Limited.

In addition to the sulfur copolymers invented in 2013 [42], Zhou et al. recently synthesized a similar low-melting-point sulfur compound (sulfur iodide molecular crystal, e.g., S_9.3_I with a melting point of ~65 °C) (Figure 5a–d) [43]. The sulfur iodide showed a much higher electrical conductivity (semi0conductive, ~5.9 × 10^−7^ S cm^−1^ at 25 °C, 11-order-of-magnitude increase) compared to pristine sulfur (S_8_) by altering the band gap in the sulfur crystal, which further expands the scope of the solid-state chemistry of sulfur. When applied as a cathode in a typical solid-state full cell, it showed a relatively high capacity of >800 mAh g^−1^, higher rate capacities (e.g., 685 and 489 mAh g^−1^ at 4.0 and 5.6 A g^−1^, respectively), and a long cycle life (87% capacity retention over 400 cycles, with high CE of 99.8%) at room temperature (25 °C) (Figure 5e,f). It is worth noting that, due to the lower melting point, the specific cathode can be thermally healable (e.g., at 100 °C) and shows something of a boost in capacity due to the melt-driven interface repair and electrolyte kinetics, which solve many of the operational challenges of solid-state LSBs (SSLSBs); also, these SSLSBs deliver higher capacity at higher temperatures (e.g., 812 and 1211 mAh g^−1^ at 0.16 A g^−1^ when increased from 25 to 100 °C). However, it should be mentioned that the mass loading of active material (40 wt.% S_9.3_I in the composite cathode) is low and the specific energy is even lower in the solid-state full-cell prototype.

#### 2.1.2. Electrolytes

**Figure 6:**
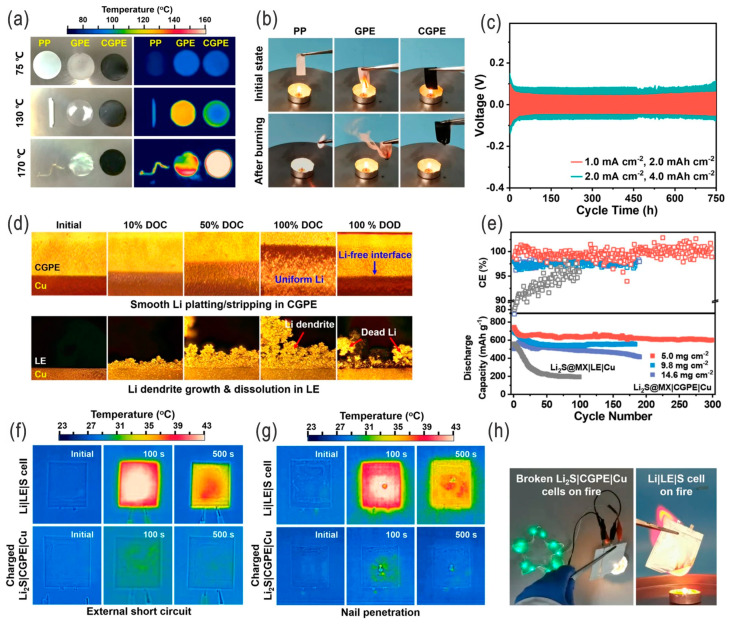
Physicochemical and electrochemical characterizations of the composite gel polymer electrolyte (CGPE): (**a**) Thermal stability tests of polypropylene (PP), MXene-free GPE, and CGPE (left: optical photos, right: infrared thermography), and (**b**) the corresponding flame tests. (**c**) Voltage–time profiles of Li|CGPE|Li cells. Performance of quasi-solid-state anode-free Li_2_S@MX|CGPE|Cu cells: (**d**) Operando optical images of Li deposition on a Cu foil surface at different stages of charge–discharge in a Li_2_S@MX|CGPE|Cu cell, compared to a Li_2_S@MX|LE|Cu full cell (MX: MXene, LE: liquid electrolyte), initially charged from OCV to 3.5 V and then discharged to 1.7 V. (**e**) Cycling stability and CE of Li_2_S@MX|CGPE|Cu cells as well as Li_2_S@MX|LE|Cu cells (at 233 mA g^−1^). Mechanical and thermal abuse tests of as-prepared quasi-solid-state Li_2_S@MX|CGPE|Cu pouch cells. (**f**,**g**) Infrared thermography of charged Li_2_S@MX|CGPE|Cu full cells, compared to Li|LE|S cells, after external short circuit and nail penetration, respectively. (**h**) Flame test of charged Li_2_S@MX|CGPE|Cu and Li|LE|S cells. Adapted with permission from Ref. [45]. Copyright 2022 the author(s). Published by Springer Nature. Open access licensed under a Creative Commons Attribution 4.0 International License.

Liquid electrolytes for LSBs mainly include carbonate-based (generally excluded due to the possible irreversible reaction with LPSs), ether-based, other organic solvent-based, ionic liquid-based, mixed, modified gel-type (e.g., by using non-flammable MXene-doped fluorinated polymers), quasi-solid-state [44,45], and solid-state electrolytes (e.g., polymer electrolytes, inorganic solid electrolytes, composite electrolytes) [46]; the choice of the relatively poorly solvating liquid electrolytes helps to minimize the polysulfide shuttle effect [47]. Electrolyte additives such as LiNO_3_ are beneficial to the passivation (i.e., protection) of the Li anode and suppress the parasitic reactions with soluble polysulfides; thus, higher CEs (up to 96–99%) can be achieved over long-term cycling (e.g., 1500 cycles) [48,49]. Solid-state electrolytes are simultaneously conducive to suppressing the lithium dendrites [4,50]. For example, Liu et al. developed a quasi-solid-state electrolyte consisting of non-flammable MXene/fluorinated polymer/LiTFSI, which simultaneously suppressed LPS shuttling and lithium dendrite formation while securing the safety of high-energy-density anode-free LSBs (against electrical, thermal, and mechanical abuses) (Figure 6) [45].

Compare to traditional carbonic ester-based electrolytes used in LIBs, the optimized electrolytes for LSBs now mainly focus on the following ether-based electrolytes [47]: lithium bis(trifluoromethylsulfonyl)imide (LiTFSI) resolves in the mixture of 1,3-dioxolane and 1,2-dimethoxyethane (DOL/DME), or glymes (polyethylene glycol dimethyl ether, PEGDME, e.g., G2, G3 and G4) [30,49,51]. That is, highly soluble electrolytes may not be the best choice, while the properly soluble electrolytes (including sparingly solvating electrolytes) for polysulfides (Li_2_S*_x_*) are more suitable with regard to the specific Li–S battery systems for higher energy densities and better cycling performance [36,52,53]. Furthermore, lean electrolyte operation (e.g., lower electrolyte-to-sulfur ratio, E/S, approaching 1 mL g^−1^)—rather than the contemporarily adopted approaches using excessive electrolyte quantities (e.g., 15 mL g^−1^), far higher than the theoretical minimum value (Min-E/S, 0.39 mL g^−1^ [24], to fully solubilize polysulfide intermediates and realize the solution-controlled precipitation–dissolution chemistry)—is also a most promising way to enhance the energy densities, shuttle control [54], and security in large-scale applications [52]. Although their further practical applicability needs further verification in LSB systems, for an acetonitrile (ACN) solvate system, an initial study revealed that the ACN electrolyte (with a 2:1 ratio to lithium salt, i.e., ACN_2_LiTFSI, LiTFSI: lithium bis(trifluoromethane)sulfonamide) possesses a comparable sulfur utilization ratio to conventional DOL/DME systems at higher temperatures (55 °C), as well as a different Li–S reaction pathway [55]; a specific quasi-solid conversion (unlike conventional dissolution–precipitation) for sulfur speciation also happens in glyme-based lean electrolytes [51].

**Figure 7:**
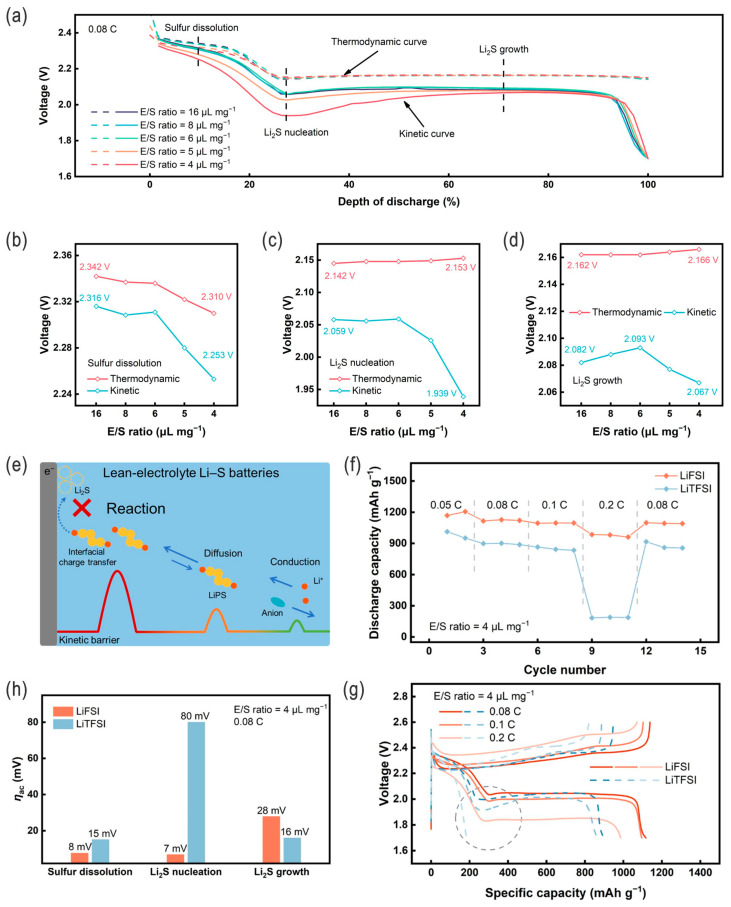
(**a**) Thermodynamic and kinetic curves of the sulfur cathode at different E/S ratios during discharge, and (**b**–**d**) the corresponding thermodynamic and kinetic voltages at the sulfur dissolution stage, Li_2_S nucleation stage, and Li_2_S growth stage, respectively. (**e**) Schematic illustration of electrode reaction processes in the sulfur cathode regarding interfacial charge transfer, reaction species diffusion (Li_2_S*_x_*, 6 ≤ *x*, Li_2_S*_y_*, 4 ≤ *x* ≤ 6), and ion conduction in the electrolyte that bring about activation polarization (*η*_ac_), concentration polarization (*η*_con_), and ohmic polarization (*η*_ohm_), respectively, by decoupling cathodic polarization (*η*_total_) during discharge. Kinetic evaluation and battery performances of the lean-electrolyte LSBs using LiFSI electrolytes: (**f**) Rate performances of LSBs at an E/S ratio of 4 μL mg^−1^, and (**g**) the corresponding charge−discharge profiles at different rates. (**h**) Decoupled activation polarization analysis of lean-electrolyte LSBs with LiFSI electrolytes, compared to LiTFSI electrolytes. Adapted with permission from Ref. [56]. Copyright 2023 American Chemical Society.

**Figure 8:**
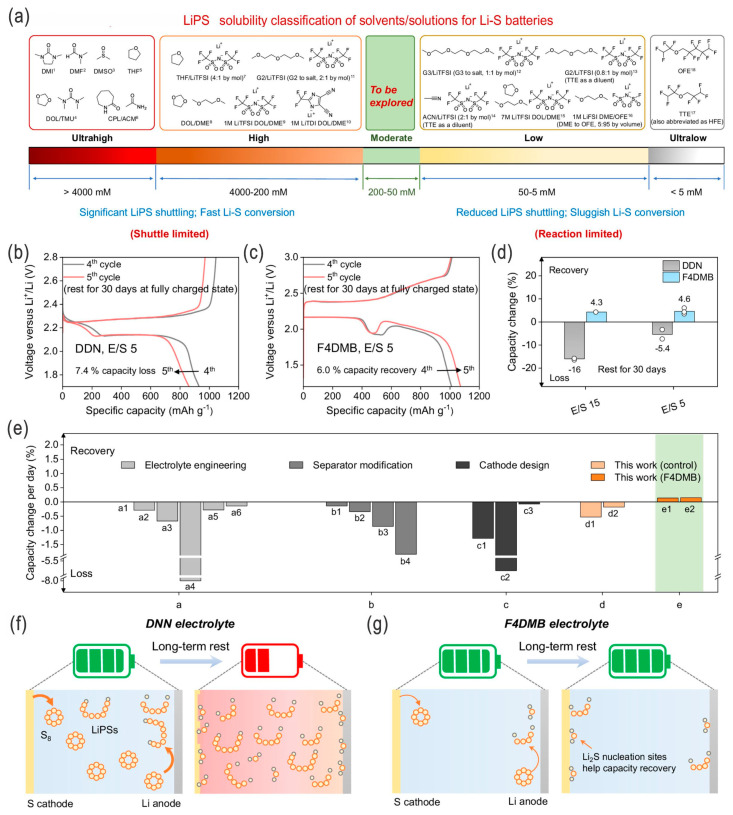
(**a**) Design concepts of high-performance electrolytes for LSBs by LPS solubility classification of solvents and solutions, i.e., electrolytes with moderate LPS solubility (50–200 mM, in S atom, [S]) are to be explored. Calendar-aging/self-discharge behavior in the two DDN and F4DMB electrolytes: (**b**,**c**) Galvanostatic charge–discharge (GCD) profiles of Li–S cells with resting protocols (30 days at fully charged state) using DDN and F4DMB electrolytes, respectively, under lean-electrolyte conditions (E/S = 5, at 0.1 C, room temperature), and (**d**) the corresponding capacity change after a 30-day long-term rest. (**e**) Capacity fade rate chart of self-discharge behavior compared with other different systems via suppression of self-discharge by electrolyte engineering, separator modification, or cathode design. (**f**,**g**) Schematic illustration of the self-discharge behavior in DDN and F4DMB electrolytes, respectively. Adapted with permission from Ref. [53]. Copyright 2023 the author(s). Published by PNAS. Open access under Creative Commons Attribution-NonCommercial-NoDerivatives License 4.0 (CC BY-NC-ND).

For example, Chen et al. studied the cathode kinetics in lean-electrolyte Li–S batteries, revealing that the activation polarization (rather than concentration and ohmic parts) was the dominant one in the cathodic polarizations with the decrease in the E/S ratio, and the resultant sluggish interfacial charge-transfer kinetics mainly degraded the cell performances (Figure 7a–e). Accordingly, this could be remediable by adopting proper electrolytes, e.g., lithium bis(fluorosulfonyl)imide electrolyte (LiFSI), to decrease the activation polarization, and a comparably high discharge capacity of 985 mA h g^−1^ at 0.2 C could be achieved with a low E/S ratio of 4 μL mg^−1^ (Figure 7f–h) [56]. Nazar and coworkers revealed that, under lean-electrolyte conditions, not only can quasi-solid state sulfur conversion be invoked, but also the lithium dendrite will be suppressed, i.e., simultaneously resolving the two critical challenges for dissolution/shuttling of super-concentrated LPSs and electrolyte depletion by the Li anode; also, the high-safety and low-flammability electrolytes are more suitable for EV applications [51]. Cui and coworkers developed a series of fluorinated organic electrolytes with moderate polysulfide solubility (e.g., 50–200 mM based on S atom, compared to ~1100 mM for DOL/DME), since the highly soluble electrolytes usually cause severe self-discharge and fast capacity decay, while the significantly low-solubility electrolytes generally have sluggish interfacial charge-transfer kinetics (i.e., high resistance in the probable solid–solid S ↔ Li_2_S electrochemistry) (Figure 8a). By using these fluorinated electrolytes, such as fluorinated-1,4-dimethoxybutane, the LSBs can demonstrate a high reversible capacity of 1526 mA h g^−1^ at 0.05 C and an outstanding CE of 99.89% can be obtained over 150 cycles at 0.2 C under the desirable lean-electrolyte conditions at 60 °C, while for room-temperature conditions the capacity is somewhat lower. That is, with an optimized LPS solubility of ~70 mM, the LSBs could deliver a 5-fold increase in cycle life compared to traditional ether-based commercial electrolytes under lean-electrolyte and high-sulfur-content conditions, and amazingly, no self-discharge was observed, but a capacity increase/recovery (by 4.3%) appeared after a 30-day aging life (Figure 8b–f) [53]. The correlation between the quantitative solubility of LPSs (lithium polysulfides), the fluorination degree of electrolyte solvent, and battery properties can thus be systematically evaluated to some extent.

It should be mentioned that there are also some studies showing paradoxical cases where high-solvation electrolytes, e.g., 1,3-dimethyl-2-imidazolidinone (DMI) [57], are beneficial for their initial high capacities, even under lean-electrolyte conditions (e.g., 5 μL_electrolyte_ mg_sulfur_^−1^); however, their long-term cycling stability is yet to be resolved. In these electrolytes, with high solubility of polysulfides, a new reaction mechanism of the activation of sulfur radicals (S_3_^•−^) further enables the high sulfur utilization [57].

**Figure 9:**
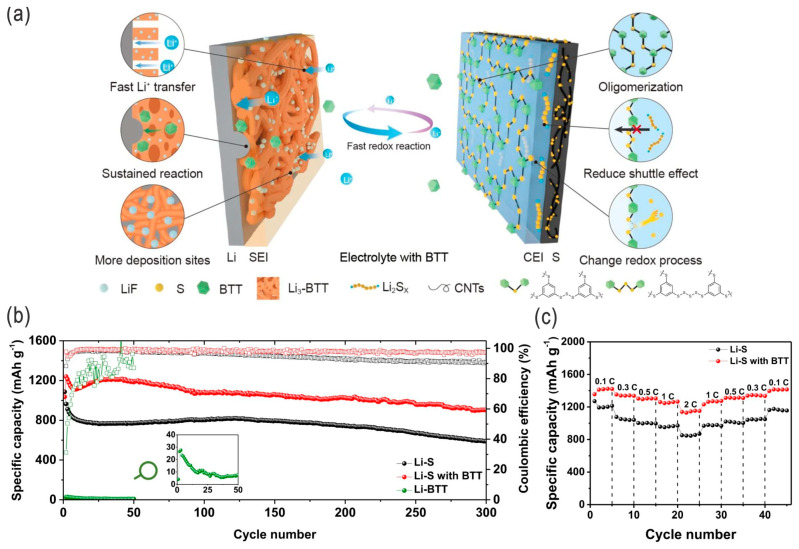
(**a**) Schematic illustration of the LSBs with the electrolyte additive 1,3,5-benzenetrithiol (BTT) for dual solid–electrolyte interfaces (D-SEIs) formed on the interfaces of the anode and cathode. Electrochemical performance of LSBs with BTT and blank electrolyte (without BTT): (**b**) Long-term cycling performance and CE at 1 C rate, and (**c**) C-rate performance. Adapted with permission from Ref. [64]. Copyright 2021 the author(s), Springer Nature, Open access licensed under a Creative Commons Attribution 4.0 International License.

Dendrite issues are not only inevitable in Li/Na metal batteries [58,59,60] but also encountered in Li–S batteries with Li metal anodes. High-fluorinated cosolvents/additives, by using the experience of Li metal batteries, can be added to the (highly concentrated) electrolytes of LSBs to suppress the formation of Li dendrites, e.g., inert fluoroalkyl ether (1H,1H,5H-octafluoropentyl-1,1,2,2-tetrafluoroethyl ether) (OFE) [61], or 1,1,2,2-tetrafuoroethyl 2,2,3,3-tetrafluoropropyl ether (HFE) [62]; conventional dimethoxyether (i.e., 1,2-dimethoxyethane, DME) can also still be used as an electrolyte solvent. These high-fluorinated and localized high-concentration electrolytes are an alternative solution for simultaneously addressing the dendrite formation and minimizing the (high-order) polysulfide solubility by using conventional ether-based electrolyte solvents. However, the high-fluorinated electrolytes have relatively higher costs and could be further optimized by using low-fluorinated solvents or by using low-cost inorganic fluoride salts/acids [63]. Some bifunctional electrolyte additives, such as 1,3,5-benzenetrithiol (BTT), can address both the interfacial instability of Li metal and the shuttle effect of LPSs via the in situ organothiol transformation, i.e., the simultaneous reaction with the Li metal anode and sulfur cathode to form respective SEI films on both electrodes (Figure 9) [64]. A Li metal protection layer may also be adopted to inhibit the Li corrosion behavior and SEI dissolution (SEI: solid–electrolyte interface film), while the artificial passivation layer (or corrosion-inhibiting layer) consisting of low-solubility metal fluorides and polymers (e.g., a MgF_2_/PVDF layer coated on Li foil or a Cu current collector) can enhance anti-corrosion and interface stability, thus suppressing the irreversible lithium loss in these lithium metal batteries [65].

**Figure 10:**
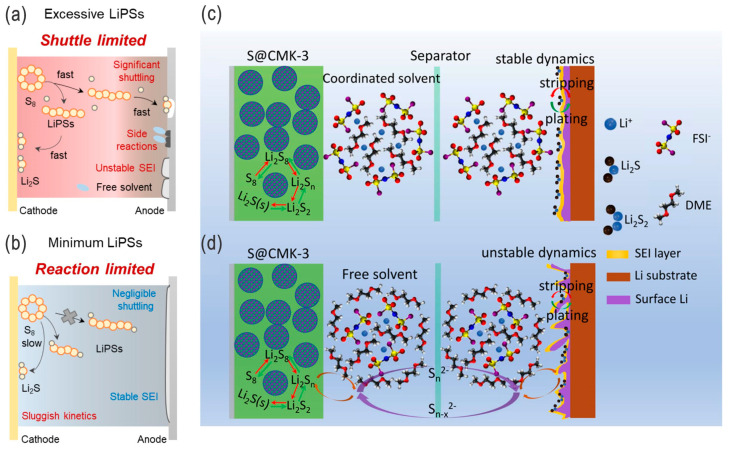
(**a**,**b**) Schematic illustrations of the working mechanisms of LSBs with ultrahigh/high LPS solubility and low/ultralow LPS solubility, respectively. PNAS 2023, [53]. (**c**,**d**) Schematic illustrations of LPS shuttling and lithium dendrites for LSBs in concentrated and diluted electrolytes, respectively. Adapted with permission from Ref. [62]. Copyright 2018 Elsevier Ltd.

Highly concentrated electrolytes are also beneficial to simultaneously suppress the dendrite growth and polysulfide shuttling, e.g., Wang and coworkers used a 12 M lithium bis(fluorosulfonyl)imide (LiFSI) salt dissolved in DME (12 M LiFSI/DME), which achieved high coulombic efficiencies for lithium stripping/plating (>99.2%) and the sulfur cathode (>99.7%) by the formation of a robust LiF-rich SEI film and controlled dendrite growth. The optimized LSBs showed high long-term stability, e.g., 786 mA h g^−1^ at 0.1 A g^−1^ and 644 mA h g at 1 A g^−1^, after 300 cycles with no detectable shuttle reactions (Figure 10) [62].

#### 2.1.3. Binders

**Figure 11:**
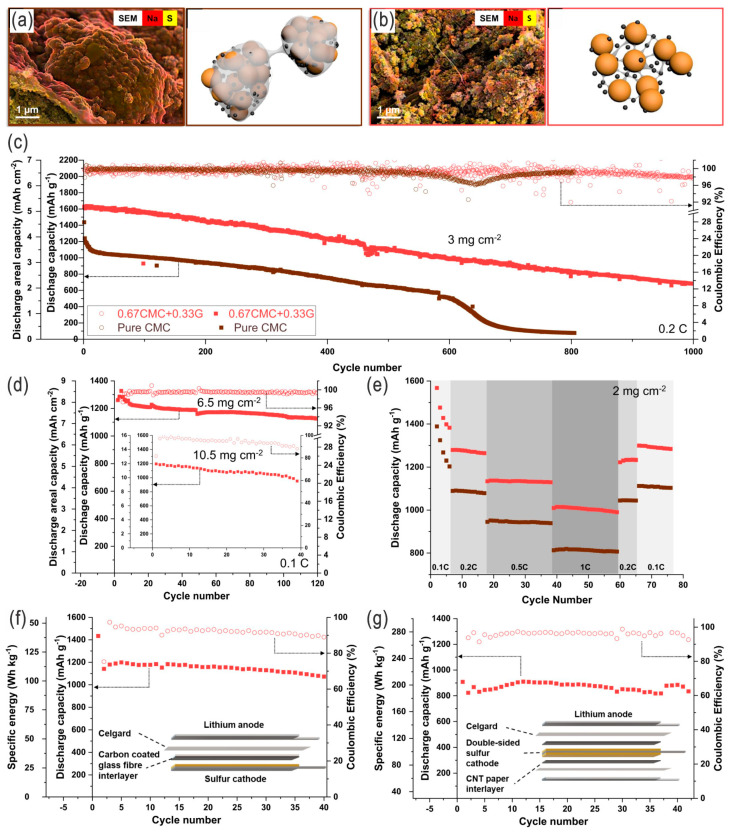
(**a**,**b**) SEM images and corresponding schematic illustrations of sulfur electrodes with different binder systems, i.e., pure carboxymethyl cellulose (CMC) and CMC/G (G: glucose). (**c**) Cycling performance of a CMC/G cathode compared with a CMC cathode in coin cells (sulfur loading of 3 mg cm^−2^), and (**d**) the corresponding performance with different sulfur loading of 6.5 and 10.5 mg cm^−2^, as well as (**e**) rate capabilities of CMC/G and CMC cathodes (red and brown; sulfur loading of 2 mg cm^−2^). (**f**,**g**) Pouch cells configured with single-sided cathodes and double-sided cathodes, respectively, in lean-electrolyte conditions (E/S = 6 μL mg^−1^), optimized for higher specific energy for practical LSBs. Adapted with permission from Ref. [67]. Copyright 2021 the author(s), Springer Nature, Open access licensed under a Creative Commons Attribution 4.0 International License.

Polyvinylidene fluoride (PVDF) and carboxymethylcellulose (CMC) are typical binders for LSBs; some more novel binder systems, such as polar/electronegative saccharides, gums, cellulose, modified cyclodextrin, CMC-SBR (copolymer with styrene-butadiene rubber), PEO (polyethylene oxide), redox-active π-stacked supramolecular polymers [66], electroactive conducting polymers, and nanocomposites (polypyrrole/polyurethane, PPyPU), have been recently explored for efficient polysulfide regulations as well as for buffering the volume change. Furthermore, elastomeric binders may also assist in further mitigating the mechanical degradation of cathodes. For example, Huang et al. exploited a glucose-based binder with the advantages of forming viscoelastic filaments during casting to improve the microstructures of sulfur cathodes (web-like), thus providing a durable sulfur cathode with minimal polysulfide escape, viz., an extremely high sulfur utilization of 97% and a high capacity retention of ~700 mAh g^−1^ after 1000 cycles in 9 months (Figure 11) [67]. However, although this binder is of much lower cost and its longevity is desirable, the assembled LSBs showed a limited specific energy of merely 206 Wh kg^−1^, which is comparable to that of LIBs and requires further enhancement for more competitive practical applications. Zhou et al. utilized an aqueous inorganic polymer multifunctional binder (ammonium polyphosphate, APP), which endowed the LSBs with not only enhanced flame-retardant properties (i.e., improved safety) but also higher electrochemical performance compared to traditional PVDF binders, due to its strong binding with LPSs and intrinsic fire resistance [68]. Another biomacromolecular binder (tragacanth gum) showed similar high performances (e.g., high rate, cycle stability, and areal capacity), superior to LSBs with PVDF or PEO binders, i.e., a 46% improvement in polysulfide entrapment, less volume change (within 16%) even at 4 C, high sulfur loading up to 12 mg cm^−1^ with no compromise in sulfur utility and reversibility, and flexible LSBs with gravimetric energy density of 243 Wh kg^−1^ [69].

#### 2.1.4. Interlayers

**Figure 12:**
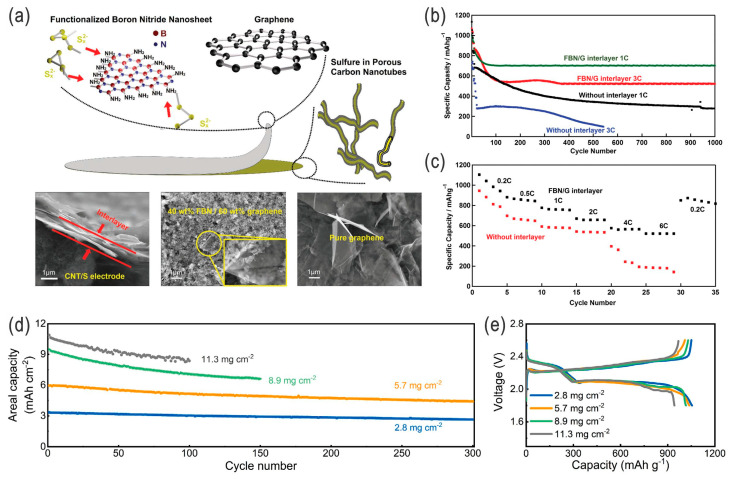
(**a**) Schematic illustration of the configuration of a Li–S cell with a functionalized boron nitride nanosheet/graphene (FBN/G) interlayer, as well as the corresponding SEM images of the cross-section and surface of the FBN/G interlayer and the surface of the pure graphene interlayer. (**b**) Ultra-long cycling performance of LSBs with and without an FBN/G interlayer (at 1 C and 3 C), and (**c**) corresponding rate performance. Adapted with permission from Ref. [72]. 2017 WILEY-VCH Verlag GmbH & Co. KGaA, Weinheim. (**d**) Long-term electrochemical cycling performance of LSBs with atomic Co–N–C electrocatalysts at different sulfur loadings, and (**e**) corresponding GCD profiles. Adapted with permission from Ref. [73]. Copyright 2019 the authors. Published by John Wiley & Sons Australia, Ltd., on behalf of UESTC. Open access under the terms of the Creative Commons Attribution License.

The separator is a crucial component in Li-ion batteries, as well as in Li–S batteries; however, the highly porous polymer membrane or glass fiber membrane can adsorb a large amount of electrolytes and/or highly soluble LPSs. Thus, the investigation of the diffusion path and distribution of LPSs in the separator will help in the rational design of the separator and thereby enhance the performance of LSBs [2]. The interlayers, usually conductive/electrocatalytic, between the cathode and separator (also called a multifunctional separator, or modified/composite separator as a whole; and sometimes between the anode and separator in conjunction with suppressing the dendrite growth) can effectively improve the cycling stability together with the rate performance and capacity of Li–S batteries, regardless of the design of the electrodes and electrolytes. These interlayers include high-surface-area thin porous conductive coatings on the separator (e.g., carbon paper [37], carbon-coated glass fiber [70], carbon/PVDF [2], CNT@cationic-polymer/rGO [71], MOF/GO), transition-metal oxides/chalcogenides (e.g., TiO_2_/PTFE), conductive or semi-conductive 2D materials (e.g., functionalized boron nitride nanosheets/graphene composites [72], Figure 12a–c), or atomic-scale electrocatalysts (Co–N–C, Figure 12d,e) [73] with lithiophilic/sulfiphilic sites, to avoid the internal accommodation and precipitation of polysulfides as an inactive S-related species layer, thus providing improved sulfur utilization and a higher specific capacity and cycling stability.

Some multifunctional interlayers, such as highly thermally conductive composite separators (LBL-assembled boron nitride nanosheets and polyacrylic acid [74]) with efficient thermal management features, can address the thermal runaway concerns (or undesirable elevated temperatures or temperature gradients). In addition to the interlayer adjacent to the sulfur cathode, carbon and its composites (e.g., Super P carbon, Ag/C) can also be inserted as interlayers between the anode (Li metal) and the separator (including solid electrolyte) to suppress the Li dendrite growth [43]. In addition, some other separators, such as biomimetic self-assembled aramid nanofiber (ANF) membranes with negative charge on nanoscale pores/channels, may demonstrate enhanced cycle life of LSBs (3500+ cycles up to 3 C rate) due to the suppressed LPS diffusion and dendrite growth compared to conventional Celgard membranes [75].

### 2.2. Reaction Kinetics

LPSs’ conversion kinetics is a critical factor influencing the sulfur utilization, rate performance, and power densities. The improvement of conductivity and the introduction of electrocatalysts are two main strategies to overcome the sluggish kinetics.

#### 2.2.1. Conductivity

Improving the sulfur utilization ratio and rate performance depends on the superior electric conductivity and the ionic transport, which are critical to the capacity and energy/power densities of the Li–S batteries. In addition to the nanoengineering of the cathode and its composition, optimized conductive skeletons are critical for the enhancement of performances, e.g., the fabrication of 3D conducing networks by using 3D porous carbon matrices/nonwovens [76,77], 1D carbon nanotubes/nanofibers [78,79], and 2D graphene or carbon nanosheets [80]. Even an integral approach could be adopted (e.g., carbon nanotubes + electrocatalysts) to enable Li–S full-pouch cells with exceptional power and energy densities [81].

#### 2.2.2. Electrocatalysis

**Figure 13:**
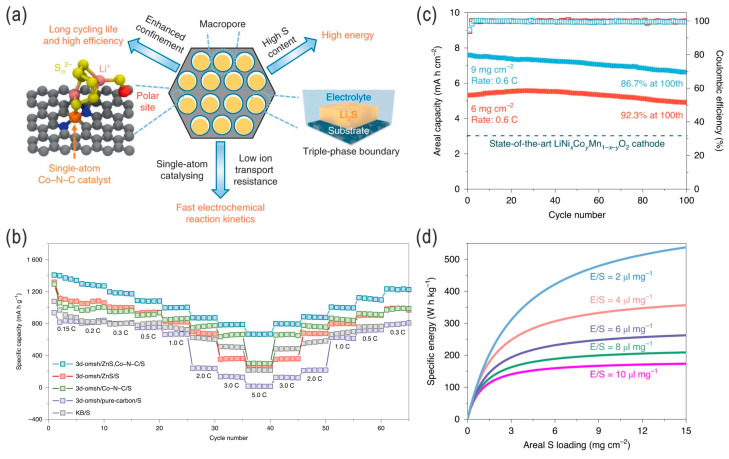
(**a**) Design strategy of the conductive catalytic cathode of a 3D ordered macroporous sulfur host (“3d-omsh” for short) with double-end binding (DEB) sites (Co–N–C SAC site and the ZnS polar site). (**b**) Rate capabilities of various sulfur cathodes. (**c**) Cycling performance (discharge capacity, along with capacity retention and CE) of the 3d-omsh/ZnS, Co–N–C/S cathode in coin-cell configuration with high sulfur loading of 6 and 9 mg cm^−2^ at 0.6 C. (**d**) Calculated specific energy vs. areal S loading for Li–S pouch cells at different E/S ratios (assuming the specific capacity and average discharge voltage are 1000 mA h g^−1^ and 2.05 V, respectively). Adapted with permission from Ref. [76]. Copyright 2020 UChicago Argonne, LLC, Operator of Argonne National Laboratory under exclusive license to Springer Nature Limited.

To improve the adsorption/immobilization and catalysis of polysulfides, and for alleviated shuttling and enhanced conversion kinetics of Li–S chemistry, the introduction of electrocatalytic polar transition-metal alloys/oxides/chalcogenides and MXenes (e.g., Co@NC Mott–Schottky heterostructures [82], CoZn clusters/carbon nanocomposites [83], N-doped Co_9_S_8_ to reduce the overpotential of commercial Li_2_S cathodes [84], Ni–Co alloy/oxide particles embedded in porous carbon nanofiber cloth [38], Ru–RuO_2−*x*_@NC hollow nanofibers [85]) is an effective strategy [86]. An optimal electrocatalyst may have the following features: high conductivity (for fast electron transfer), high LPS adsorption capability, and high catalytic efficiency (for LPS conversion reactions). Qiao and coworkers developed a kind of high-power (or fast-charging) LSB via rational electrocatalyst engineering of a transition-metal/carbon nanocomposite. The CoZn clusters embedded in the carbon (rGO) nanocomposite electrocatalyst demonstrated enhanced SRR kinetics, conducive to the achievement of long-lifespan and high-power LSBs, e.g., 1000 cycles at 8 C (current density of 13.4 A g^−1^ with 5 mg cm^−2^ sulfur loading) and high capacity retention of ~75% (500 mAh g^−1^ for the final discharge process), corresponding to an initial specific energy of 1.3 kWh kg^−1^ and a high specific power of 26.1 kW kg^−1^ (fast charge/discharge, <5 min) [83]. As an updated version, the introduction of anchoring sites (i.e., polar materials by polar–polar interaction with Li^+^, and single-atom catalysts (SACs) [87] such as Fe–N–C or Co–N–C by Lewis acid–base interaction, which binds the center metal atom toward S_n_^2−^) into the sulfur hosts is an emerging and effective strategy to immobilize LPSs and enhance the redox kinetics for the regulation of the shuttle effect, improved sulfur utilization, and high-rate performance [76]. Zhao et al. designed a highly oriented macroporous catalytic cathode with double-end binding sites (i.e., polar and uniformly dispersed ZnS nanoparticles and a Co–N–C single-atom catalyst) via the thermal treatment and nanocompositing of a colloid-templated Zn,Co-containing zeolitic imidazolate framework (ZIF), which showed advantages in the immobilization and catalytic conversion of polysulfide intermediates (LPSs) during repeated charge–discharge processes, viz., suppressed shuttle effect and Li metal corrosion, which boosted the sulfur utilization and redox reaction kinetics (Figure 13a). The enhanced ionic transport, even at high sulfur loading and/or lean-electrolyte conditions, avoids the occurrence of inactive/dead sulfur, thus providing an improved cell-specific energy of >300 Wh kg^−1^ with coulombic efficiency of >95% in a 1-Ah-level pouch cell with just 100% lithium excess (Figure 13b–d) [76].

Nazar’s group recently reported a metallic interfacial redox mediator (LiVS_2_) coated Li_2_S particles for high-performance all-solid-state LSBs by improving the cathode conversion kinetics [88]. With the conventional superionic argyrodite-type solid electrolyte, the modified Li_2_S cathode showed good rate capabilities up to 3 mA cm^−2^ and high capacity retention of around 80% over 1000 cycles at 1 mA cm^−2^ and room temperature, as well as high CE (close to 100%) and high areal capacities (e.g., 5.3 mAh cm^−2^) with high active material loading (e.g., 10 mg cm^−2^) at 60 °C. However, the sulfur utilization ratio should be increased at higher active material loading for cost-effective practical applications. Manthiram’s group developed similar well-mixed Li_2_S–TiS_2_ composite cathodes with conductive TiS_2_ as a functional catalyzing agent to reduce the activation energy barrier and improve the activation efficiency and Li_2_S utilization, although a classic ether-based liquid electrolyte and integrated carbon-paper current collector were applied [89].

### 2.3. Anodes

Due to the capacity limit of the current commercial insertion-type anodes, e.g., graphite (~372 mAh g^−1^) for lithium-ion batteries or Li metal batteries using extremely high capacity (ten times higher, theoretically 3860 mAh g^−1^), the sufficiently low density and electrode potential of Li metal anodes make them the most promising type of energy storage devices [90]. However, dendrite-growth-related low coulombic efficiency and safety problems generally exist in Li metal batteries (LMBs), including LSBs. Learning from the experiences of LMBs [8], several valid approaches can be utilized to address the dendrite growth [10,91], e.g., electrolyte additives, fluorinated electrolytes [16], solid electrolytes, hierarchical lithium hosts (micro/nanostructured) [10], (modified) porous lithium electrodes [92], and artificial protective films (polymer, inorganic, organic–inorganic, alloy, or artificial SEI) [90].

**Figure 14:**
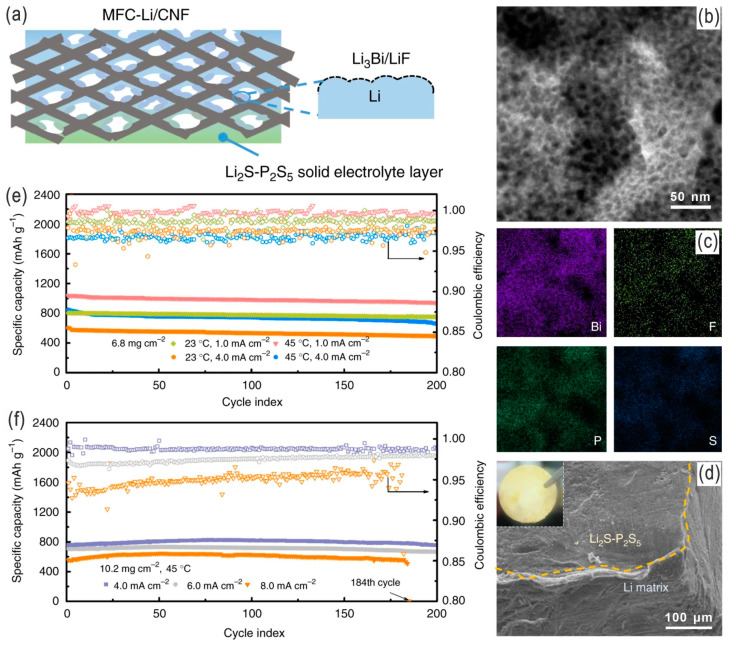
(**a**) Schematic illustration of a coating strategy to derive a porous metal–fluoride complex protected Li/carbon nanofiber (MFC-Li/CNF) electrode, i.e., coating Li with solubilized BiF_3_. (**b**,**c**) TEM image and EDS analysis, respectively, of the protective layer stripped from the MFC-Li electrode. (**d**) SEM image of the edge of the MFC-Li/CNF electrode. Cycling performance of LSBs employing MFC-Li/CNF electrodes in different conditions: (**e**) sulfur loading of 6.8 mg cm^−2^ at 1.0 and 4.0 mA cm^−2^ (at 23 and 45 °C); (**f**) sulfur loading of 10.2 mg cm^−2^ at 4.0, 6.0, and 8.0 mA cm^−2^ (at 45 °C). Adapted with permission from Ref. [92]. Copyright 2019 the author(s). Published by Springer Nature. Open access licensed under a Creative Commons Attribution 4.0 International License.

**Figure 15:**
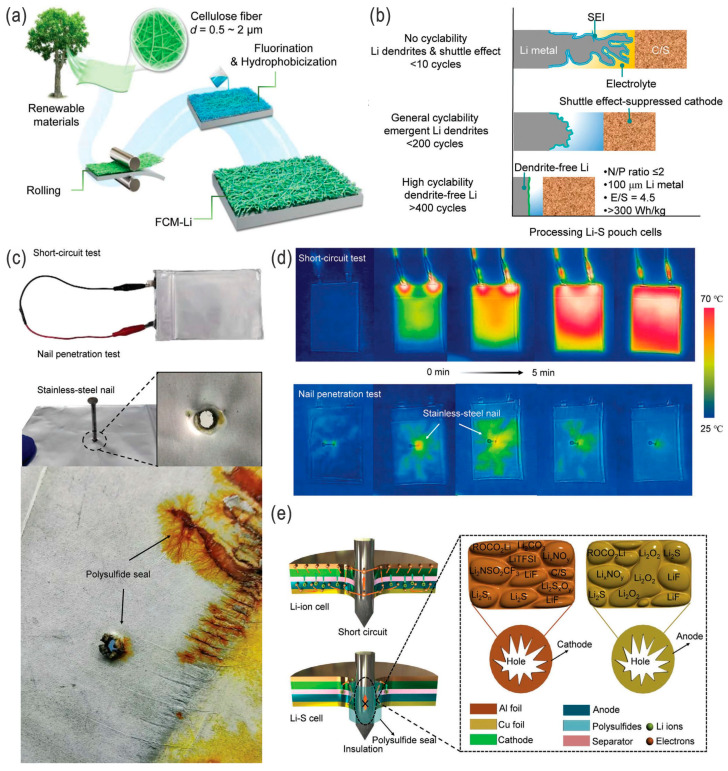
(**a**) Schematic illustration of large-scale preparation of a functionalized anode (FCM-Li) by roll-pressing a sustainable cellulose membrane on its surface. (**b**) Schematic illustration of Li metal anodes tailored using different strategies for processing Li–S pouch cells. Safety demonstration: (**c**) Optical images of short-circuit and nail penetration tests. (**d**) IR images of Li–S pouch cells during short-circuit and nail penetration tests. (**e**) Schematic illustration of the cross-section structures of nail-penetrated Li-ion and Li–S cells, as well as the chemical composition of the cathode and anode at the puncture region. Adapted with permission from Ref. [93]. Copyright 2024 Wiley-VCH GmbH.

Ren et al. fabricated a porous lithium electrode on an interwoven carbon nanofiber (CNF) scaffold with a Li_3_Bi alloy/LiF composite coating layer (with high ionic conductivity of estimated 6.9 × 10^−4^ S cm^−1^) by spontaneous reaction between Li metal and BiF_3_; on the metal–fluoride complex protected Li electrode (MFC-Li), a solid Li_2_S–P_2_S_5_ electrolyte layer was synergistically formed (Figure 14a–d). This protected the porous Li anode (MFC-Li/CNF anode, compared to a bare Li or MFC-Li anode), endowing the LSBs with carbon cloth/sulfur cathodes with a higher capacity and a more stable performance with a high sulfur loading of 10.2 mg cm^−2^ at 6 mA cm^−2^ over 200 cycles (without LiNO_3_ additive) (Figure 14e,f) [92]. Chen et al. developed a sustainable cellulose membrane roll-coated Li metal plate anode, followed by fluorination and related chemical modification for hydrophobic features (Figure 15a). The as-prepared large-scale Li–S pouch cells on a pilot production line in a relatively undemanding environment (relative humidity RH = 1.55% or >300 ppm H_2_O, similar to that required for manufacturing LIBs) had an ultra-long cycling life (over 400 cycles), high average CE (99.55%), and excellent rate performance, as well as high energy and power densities (417 Wh kg^−1^ and 2766 W kg^−1^, respectively), able to power a 3 kg unmanned aerial vehicle for a long flight time. Furthermore, the as-assembled LSB showed high safety by puncture and was in fact safer than the traditional LIBs, and no rapid heat release was observed, owing to the quick passivation of the electrode and needle by the polysulfides. Also, the FCM-Li anode showed suppressed thermal runaway, as revealed by in situ infrared thermography (Figure 15b–e) [93]. It is worth mentioning that the protection or passivation of the Li metal anode by forming a protective/passivation layer not only avoids/alleviates the dendrite growth but also effectively addresses the polysulfide dissolution and mossy growth of Li dendrites (i.e., parasitic reaction) on the Li metal anode, thus inhibiting the (redox) shuttle effect [47].

## 3. Assessments

Generally, several key goals, including cost (USD ≤100 per kWh), energy densities (≥400 Wh L^−1^ and/or 400 Wh kg^−1^), and long-term cycling performance (≥1000 cycles with 80% capacity retention), are suggested to be met for these commercially applicable battery packs [52,94]. A 50 kWh Li–S battery pack with the potential to achieve 150,000 km and higher practical specific energy over 550 Wh kg^−1^ has been defined and studied in promising next-generation EVs, which are now showing 80% state of health (SoH) after 300–500 cycles and are a promising second life option for EVs compared to conventional LIBs [95]. Overall, high active material loading, less electrolyte usage, and optimized cell/battery design are desired for higher energy densities and lower costs in competitive practical application. Specifically, several parameters need to be met [4,96], e.g., high sulfur loading of >5 mg cm^−2^, low carbon content of <5 wt.%, lean electrolytes (i.e., electrolyte-to-sulfur ratio E/S < 5 μL mg^−1^, electrolyte-to-capacity ratio E/C < 5 μL per mAh), low negative-to-positive capacity ratio (N/P < 5), and high longevity (>1000 cycles) in pouch-type cells. A list of recommended pouch-cell parameters for practical application has been provided by Manthiram’s group for reference (Table 1) [97]. These parameters can be relatively easily achieved separately due to the significant progress in recent years, albeit not altogether. More efforts should be made toward the success of the eventual practical applications.

### 3.1. Characterizations

The direct visual observation of the polysulfide-trapping effect can be conducted in an H-type cell under an inert atmosphere (e.g., Ar), in which the two glass cells filled with polysulfide (e.g., Li_2_S_6_) solution and pure solvent at the same level are separated by the interlayer (sometimes supported by a separator/framework if necessary) [98]. The combined electrochemical method (i.e., EIS–GITT polarization decoupling method) using electrochemical impedance spectroscopy (EIS) and the galvanostatic intermittent titration technique (GITT) could be used to decouple the cathodic polarization and the cathode kinetics evaluation, which is of great help for Li–S batteries, especially under lean-electrolyte conditions [56]. In operando or in situ X-ray diffraction (XRD), X-ray microscopy (XRM), X-ray tomography (XRT), UV–vis, nuclear magnetic resonance (NMR) spectroscopy, infrared spectroscopy (IRS), Raman spectroscopy, optical microscopy (OM), and transmission electron microscopy (TEM) are useful spectroscopic/visualization techniques to reveal the chemical and structural evolution mechanisms [10,99,100]. Specifically, synchrotron X-ray absorption spectroscopy (XAS) measurements and molecular orbital (MO) calculations can reveal the role of orbital occupancy in electrocatalysts to determine/predict LPS concentrations and critical sulfur reduction reaction (SRR) kinetics, thus being beneficial to a direct and rational design of high-power LSBs [83].

### 3.2. Cost-Effectiveness

Gravimetric/volumetric energy densities are a key indicator for the cost-effective and practical application of Li–S batteries; high mass loading, (extremely), lean electrolytes, etc., are typical solutions to achieve these requirements. Power densities are of critical importance for their eventual practical application in EVs with higher power requirements. For high-energy-density LSBs, excessive electrolyte will dramatically degrade their advantages, and a lean electrolyte pathway is essential to achieve the goals. Although the sulfur redox kinetics favors excessive electrolyte (i.e., low lithium polysulfide concentration), and the low E/S ratio makes the sulfur cathode kinetics extremely sluggish and leads to reduced discharge capacity and deteriorated capabilities, significant progress has been made toward solving these problems by using highly efficient electrocatalysts, redox mediators, sparingly solvating electrolytes, and diluted electrolytes. For practical pouch cells, high energy densities of up to 700 Wh kg^−1^ have been achieved with a super-low E/S ratio of 1.7 (1.7 g of electrolyte per 1 g of sulfur), while most common cases are over 400 Wh kg^−1^ with an E/S ratio of ≤3.0 [56]. The design of low-porosity sulfur cathodes with minimized pore-filling electrolytes may be another viable strategy to improve the practical energy density of LSBs, although more research should be carried out on the mechanism of low tortuosity and the design of pores/channels and secondary structures [101].

Biochar and (extremely) lean electrolytes are effective choices for the cost-effective fabrication of LSBs. Zhong et al. utilized a biomass-derived dense Mn-doped carbon cathode host (porosity of 0.4); owing to the low-cost biochar, high mass loading (6.5 mg cm^−2^), extremely lean electrolyte (2.5 μL mg^−1^), and the high specific energy (422 Wh kg^−1^) thereof, the estimated cost of sub-Ah level pouch cells could reach as low as USD 60–90 per kWh (initial 20 cycles), less than the USD 100 per kWh of typical commercial LIBs, thus offering an opportunity for highly specific LSBs, although their volumetric energy density (446 Wh L^−1^) is not quite competitive with these of state-of-the-art LIBs [24]. Some more similar works on biomass have also investigated porous carbon matrices as anodes, together with electrocatalytic components and 1D or 2D carbon additives to the cathode; the integral approach endows the Li–S full-pouch cells with high energy densities as well as unprecedented power densities [81]. Zeta Energy (Corp., Houston, TX, USA) utilizes sulfur and natural gas feedstocks to synthesize sulfurized carbon as cathodes; combined with a novel 3D Li metal anode architecture, stable, safe, and lower-cost LSBs can be manufactured for fast-charging EV batteries, even at low temperatures [102].

### 3.3. Battery Management and Modeling

Battery modeling and state-of-charge (SOC) or depth-of-discharge (DOD) estimation methods play a critical role in the accurate prediction of EVs’ range and their performance in grid storage, as well as in the safe charge/discharge and optimal usage of batteries. Somewhat different to the incumbent LIB technologies with mature modeling methods, including mathematical models, (high-fidelity) electrochemical models, and electrical equivalent circuit models, LSBs are more suitable for using reduced-order (simplified) electrochemical models and equivalent circuit models due to their faster capacity fade and shuttling effect [103]. Although simple and computationally fast models of LSBs have not been reported/established, equivalent circuit network models (ECN, or electrical circuit modeling, e.g., multi-temperature state-dependent nonlinear polynomial-based battery models [104]) for LSBs have been established for better battery management in complex and scaled-up applications, including grids and EVs [105].

For state-of-charge (SOC) observability analysis or voltage prediction at different discharge rates/temperatures, the LSBs are relatively poor (in the range of 20–70%) because of their flat OCV–SOC (OCV: open-circuit voltage) curve feature, which makes it more challenging to estimate the SOC of LSBs than that of LIBs, but this could be improved based on real-time battery model parameterization (e.g., some constantly decreased indices, independent of external factors related to the application environment, sometimes make the behavior of LSBs more predictable [95]) [106], electrochemical impedance spectroscopy (EIS) modeling [107], or some new frameworks consisting of online battery parameter identification in conjunction with a trained estimator based on an adaptive neuro-fuzzy inference system (ANFIS) and coulomb counting [108]. Thus, a mean error of 4% and a maximum error of 7% for SOC estimation could be achieved in a realistic driving scenario [108], and through reinforcement learning and energy management, plug-in hybrid electric vehicles (PHEVs) could reduce fuel consumption by up to 14.63% and LSB degradation by up to 82.37% [109].

### 3.4. Environmental Adaptability

Wide-temperature adaptability is necessary for the broad application of LSBs in EVs and grid energy. Sub-zero and high temperatures (up to 60 °C) can be achieved by using solid-state electrolytes or cathode/separator modification (e.g., molecular layer deposited alucone coating in traditional LiPF_6_–carbonate electrolytes [110]; Fe/Ni-N@NC modified separators [111]). Via in situ/operando mechanism studies and electrolyte design including solvent and lithium salt optimization/modification, additive adoption, and alternating solid-state electrolytes, sub-zero temperature electrolytes could endow the LSBs with viability for working from high temperatures to low temperatures of −40 °C [99]. It should be noted that the sulfur in the cathodes of LSBs is in fact flammable and poses safety concerns, especially for large-scale application in EVs or grid energy storage. Some strategies have been proposed to address this problem, e.g., by using halogen-free flame-retardant sulfur copolymers (intrinsic flame retardant by introducing eugenol phosphazene monomer, 10 wt.%) [35] or biomacromolecular binders (tragacanth gum) [69].

Life-cycle assessment (LCA) of LSBs for EVs by using the ReCiPe method indicates that LSBs are more environmentally friendly compared to conventional LIBs (e.g., NCM–graphite battery packs, NCM: nickel–cobalt–manganese)—i.e., 9–90% lower impact compared to current LIBs in most categories, including the impact of global warming potential (GWP)—and are the cleanest batteries in the use stage, which could further meet the USABC (United States Advanced Battery Consortium) goals by optimizing the LSBs, such as by adopting binder-free electrodes, energy-efficient processes, decay rate improvement, and battery recycling [95,112,113,114,115]. Meanwhile, for stationary energy storage (grid) applications, LCA shows that the specific energy and the type of electrolyte salt are two major factors in reducing cradle-to-gate impacts, and the electricity source/loss, cycle life, and specific energy are the most important parameters in the cradle-to-grave scope; furthermore, the hydrometallurgical recycling of LSBs could further reduce the mineral impact, but not necessarily the environmental impacts [9]. Overall, LSBs are a good alternative compared to LIBs in terms of environmental sustainability, which makes them more promising in EVs and green grid applications.

## 4. Applications

**Figure 16:**
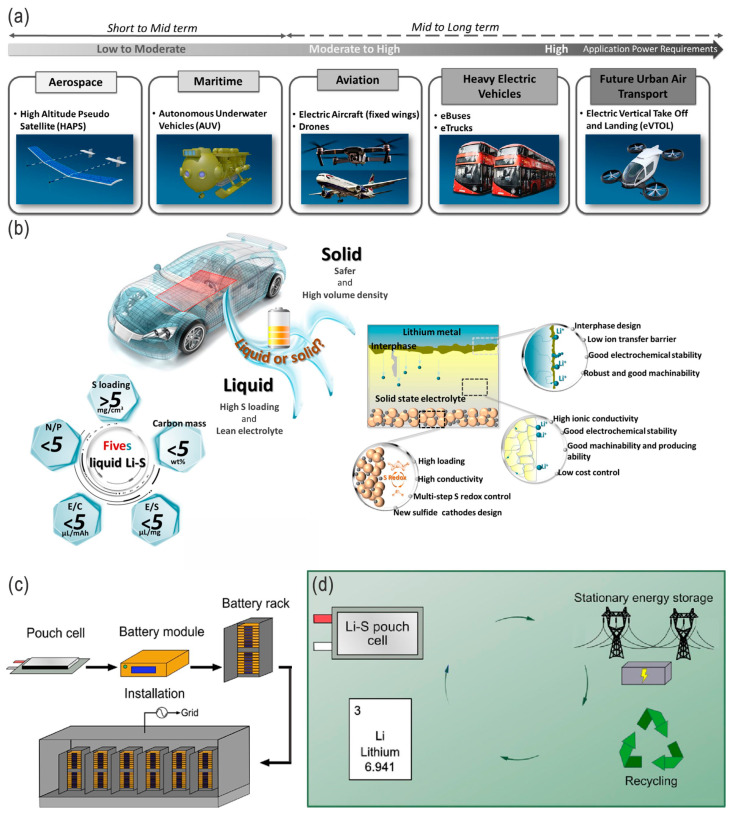
(**a**) Schematic illustration of the main emerging application areas/markets suitable for Li–S battery technology now and in the future. Adapted with permission from Ref. [136]. Copyright 2020 the authors. Published by Wiley-VCH GmbH. Open access under the terms of the Creative Commons Attribution License (CC BY 4.0). (**b**) Schematic illustration of a roadmap and metrics in the development of LSBs toward commercialization (e.g., EVs), with critical metrics for liquid LSBs and the main challenges/requirements for high-energy-density all-solid-state LSBs. Adapted with permission from Ref. [4]. Copyright 2022 the authors. Published by UESTC and John Wiley & Sons Australia, Ltd. Open access under the terms of the Creative Commons Attribution License. (**c**,**d**) Schematic illustration of LSBs (pouch cells → battery modules → battery racks → installation; inverters and fire suppression system not shown in the figure) for stationary energy storage. Adapted with permission from Ref. [9]. Copyright 2023 the authors. Published by the American Chemical Society. Open access licensed under CC-BY 4.0.

For the current most critical/challenging energy storage applications, including automobile and stationary energy storage batteries, Li–S batteries have been preliminarily evaluated with respect to their energy densities, cost, safety, lifespan, self-discharge, and environmental tolerance [116]. The market share of EVs is significantly increasing due to the increased interest in reducing CO_2_ emissions, along with economic considerations (Figure 16a). The evolution of battery capacity is a principally decisive factor for the EV market’s development. Since LIBs are reaching their theoretical limit, LSBs are considered to be a promising electrochemical energy storage system for EVs due to their advantages from the points of view of specific energy, safety, and environmental and economic issues, although the main drawbacks in durability, self-charging, power density, and battery modeling are being overcome for the possible substitution of LIBs in the near future [117]. Closed-loop modeling indicates that the LSBs hold great promise for EV applications, although they have larger internal resistance and, thus, more obvious oscillation of output voltage, as well as higher energy/power loss and relatively slow charging, compared to LIBs [105]. According to primary testing for EVs, although Li–S batteries show higher energy consumption (e.g., 17.2 kWh compared to 14.7 kWh for LIBs per 100 km), their low weight, low price, and high energy storage capacity, among other general parameters of electric batteries/EVs compared to other batteries (e.g., Na–NiCl_2_, Li-ion, Ni–MH), are showing great advantages for further EV systems [118]. As a type of new and promising renewable energy storage device, LSBs show only small or no differences compared to LIBs when cooperating with the same photovoltaic (PV) array, boost converter, and inverter in several chosen performance metrics, including voltage/current vs. time [119]. Thus, LSBs and related research methods/simulations from LIBs are suitable for photovoltaic electric vehicles (PV-EVs), EVs, PV grids, utility grids, or PV standalone/hybrid systems.

Sion Power Corporation was once a pioneer in advancing the commercialization of Li–S batteries; in 2008 it nightly powered the Zephyr, a solar/battery unmanned aerial vehicle (UAV; 18-m wingspan, 30 kg dead load and 450 kg potential payload), by using rechargeable LSBs with double the energy density of lithium polymer batteries, for a world endurance record (non-stop for more than 82 h, with a maximum operating altitude of over 18 km) [120], and in 2014 its high-energy-density cells (350 Wh kg^−1^) powered a high-altitude pseudo-satellite (HAPS) aircraft prototype of the Airbus division; however, the corporation later (in 2015) made the leap from LSBs to next-generation Li metal batteries (not those of the 1980s), with the desire to overcome or find a better balance of their uncompetitive volumetric energy density (Wh L^−1^) and cycle life for a broader market [121]. Some prototype LSBs for vehicles, such as scooter batteries and rack-mounted battery systems, have been developed by ambitious companies in recent years, including the British firm OXIS Energy (claiming its first generation of qua-solid-state LSBs with a high specific energy of 450 Wh kg^−1^ at an energy density of 550 Wh L^−1^—higher than today’s top cells of 250–300 Wh kg^−1^ [122]; however, OXIS Energy went into administration in 2021), the startup OXLiD [123], the Japanese company Sony, and the German company Theion. Although the UK-based OXIS Energy company once set forecast targets of 600 Wh kg^−1^ and 900 Wh L^−1^ as well as up to 60% weight reduction compared to conventional LIB battery systems by using their solid-state Li–S cell technology by 2026 [124], their ambitions suffered severe setbacks, and the company later went bankrupt after failing to secure fresh investment [125]. Some companies, such as Lyten (San Jose, CA, USA) in 2023, have set up pilot production lines with an output of about 100 batteries per day [126]. An Australian battery technology company, “Li–S Energy”, announced a 20-layered battery cell in 2023 by using the third-generation semi-solid-state Li–S battery (GEN3 Li–S) technology, which shows a 45% improvement in volumetric energy density (540 Wh L^−1^), higher gravimetric energy density (>400 Wh kg^−1^), and enhanced safety with the adoption of a low-flammability electrolyte. The Li nanomesh technology suppresses the dendrite formation, and the interlayer of boron nitride nanotubes (BNNTs) alleviates the polysulfide shuttle effect. Together with the optimized cathode material, with lower porosity and improved composition, the new GEN3 Li–S batteries can offer higher energy densities (roughly double the energy densities of Li-ion batteries) and enhanced safety. Commercial samples are being commenced for high-value sectors, including drones and eAviation. Full-solid-state batteries are also progressing under a joint project at Deakin (Figure 16b) [127,128]. Some novel anode engineering strategies, such as 3D lithium metal anode architecture (i.e., lithiated vertically aligned carbon nanotubes), have also been designed by companies (e.g., Zeta Energy) to avoid dendrite formation as well as to enable high charge rates and long-term stability. The novel anode shows a typical gravimetric capacity of approximately 1300–3300 mAh g^−1^, close to that of pure Li metal, but with no dendrite issues. The as-assembled LSBs can achieve a high energy density of 450 Wh kg^−1^, high rate performance (with charge rate up to 10 C), long-term cycling (2000 full charge–discharge cycles), and low self-discharge (<5% after one year) [102,129].

Batteries will play an increasingly important role in modernizing energy grids, in which they endow a higher penetration of renewable energy and better match supply with demand via overcoming intermittency and variability. However, the application of storage technology is a business activity, and its economic viability should be determined for sustainable development, viz., cost-effective battery storage is a prerequisite or essential condition [130,131]. Batteries such as LIBs and LSBs are targeting grid energy storage, including grid balancing and arbitrage (especially when integrated with renewable energy sources), as lithium costs are tumbling, and more recent advancements in battery technology as well as the help of clean energy policies and incentives are accelerating the growth and applications of renewable power and have been reinventing the green-energy landscape [130,132]. The cost advantage of Li–S chemistry makes the commercialization of grid energy storage (stationary) more achievable than EVs (automotive) after moderate improvements, since the cost control is much more crucial in large-scale energy storage [116]. For these grid-scale applications, technological and economic valuations of battery energy storage should be combined, and critical trade-offs between battery chemistry and applicability should be considered in realistic operations with different requirements/conditions for accurate revenue measurement (Figure 16c,d) [130]. Li–S flow batteries, another emerging kind of redox flow batteries (RFBs, also called polysulfide redox flow batteries (PSRFBs)), are another candidate for a low-cost, safe, and easily scalable system for grid-scale energy storage beyond the static LSBs [14,133,134]. With a scalable catholyte reservoir, they show great advantages due to their low cost and large scale; although their capacity and voltage efficiency is high (1142 mAh g^−1^ and 86.9% in flow cells), their cycle stability and lifespan need further improvements for large-scale practical applications [14]. The optimization of ion-exchange membranes (IEMs, compared with electrodes) is one of the effective strategies to mitigate the LPS shuttling and improve the coulombic efficiency [135].

## 5. Conclusions and Perspectives

Due to their great advantages of high specific energy, low cost, and environmental friendliness, LSBs have gained tremendous interest, spanning from the academic to industrial fields; however, the polysulfide shuttle effect, low conductivity (requiring extra conducting agents), volume expansion, and larger electrolyte requirements are critical issues that must be adequately addressed for their eventual large-scale practical applications.

1. The nanoengineering and nanocompositing of cathodes are of great importance to improve the capacity, energy density, and power density of the full cell. For thick cathodes with desirably high active material loading, 1D carbon nanotubes are superior to carbon black particles due to their enhanced electrical and mechanical properties. Bipolar electrodes with active materials on both sides, rather than unipolar electrodes, directly increase the energy density of the LSBs, although better adhesion properties are required during the cell assembly.
2. The introduction of interlayers is a strategy to suppress the polysulfide shuttling effect during cycling; however, these interlayers add weight to the cell and, thus, reduce the practical energy densities. Decreasing the thickness of the interlayers or increasing the functionalities of the interlayers are alternative solutions for a higher practical specific energy, e.g., via coating the composite cathodes or introducing redox-active interlayers, or compositing with separators and/or cathodes for an integrated subsystem. Introduction of the interlayer on the lithium metal anode side is a critical alternative solution to address or suppress the dendrite issue, where the nucleation and growth of lithium are controlled and the soft short-circuit thereof is avoided. However, for large-scale energy storage systems, rigid inorganic interlayers are not versatile, and flexible polymeric or gel-type interlayers, whether all-solid-state or not, are more applicable for these applications, including EVs, stationary energy storage, and smart grids.
3. Lean electrolytes are critical to elevate the energy densities; (extremely) high-concentration electrolytes (e.g., >10 M) and conventional diluted electrolytes (e.g., 1 M) with appropriate fluorides as additives/cosolvents are also alternative strategies. For the high-concentration electrolytes or fluorinated electrolytes, the polysulfides’ dissolution/shuttling and/or the lithium dendrite issue can be effectively alleviated. However, the ultrahigh concentration or high-fluorinated electrolytes/additives will increase the cost of the batteries and, thus, undermine their competiveness. Overall, they should be balanced, and moderate-fluorinated additives may be a more promising candidate.
4. LSBs will be safer for EVs than conventional LIBs; owing to the elimination of oxygen from metallic oxide cathodes and the thermal runaway thereof, their cycle lifetime might not be as long as that of conventional LIBs. Although LSBs show higher specific energy than state-of-the-art EV cells, there are many other important factors, such as power (output and charging) and cycle life, which are not sufficiently on par with them.
5. For large-scale batteries, the lithium plate or foil may not quite be applicable to achieve controlled lithium nucleation and growth to avoid the dendrite problem as well as to achieve a higher utilization ratio of active materials (e.g., lithium and sulfur) in the topological space, mesh, or (porous) carbon cloth/paper. In ideal conditions, embedding active materials such as sulfur, Li_2_S, LPSs, and Li in porous carbon cloth/paper sandwiched by polymer-type electrolytes may be the optimal strategy for eventual practical LSBs with high energy/power densities, high cost-effectiveness, scalability, and flexible properties.
6. Although LSBs are relatively eco-friendly and safer compared with LIBs, under severe conditions, such as thermal runaway and explosion, LSBs will release more toxic SO_2_ gas, in addition to the organic pollutants from electrolytes. All-solid-state LSBs that solve both the LPS shuttling and dendritic growth issues would be the ideal choice for EVs and other transport systems as well as grid storage in the future, while in the near future electrodes/electrolytes with high sulfur loading and lean electrolytes in liquid-type LSBs will be more feasible due to their low cost and high energy density.

## Figures and Tables

**Table 1:** A recommended table of critical parameters for a practically viable pouch cell.

Sulfur Content (wt.%)	Areal Sulfur Loading (mg cm^−2^)	Electrode Size (cm^2^)	E/S Ratio		
≥70	≥6.5	≥38.9	≤5		
Lithium thickness (μm)	Areal lithium loading (mg cm^−2^)	Electrode size (cm^2^)	N/P ratio		
≤50	≤2.67	≥38.9	≤1.59		
C-rate	Reversible capacity (mAh g^−1^)	Retained capacity (mAh g^−1^)	Cycle No.	Capacity retention (%)	CE (%)
0.1 C	≥1000	–	–	–	–
0.2 C	≥800	≥650	≥200	≥81.25	≥98

## Data Availability

Not applicable.

## Abbreviations

1D/2D/3D	One/two/three-dimensional
CE	Coulombic efficiency
CNTs	Carbon nanotubes
LIBs	Lithium-ion batteries or Li-ion batteries
LPSs	Lithium polysulfides
LSBs	Lithium–sulfur batteries or Li–S batteries
NC	Nitrogen-doped carbon
OCV	Open-circuit voltage
PVDF	Polyvinylidene fluoride
rGO	Reduced graphene oxide
SAC	Single-atom catalyst
SEI	Solid–electrolyte interface
SSLSBs	Solid-state Li–S batteries

## References

[1] Gür T.M. (2018). Review of electrical energy storage technologies, materials and systems: Challenges and prospects for large-scale grid storage. Energy Environ. Sci..

[2] Yao H., Yan K., Li W., Zheng G., Kong D., Seh Z.W., Narasimhan V.K., Liang Z., Cui Y. (2014). Improved lithium–sulfur batteries with a conductive coating on the separator to prevent the accumulation of inactive S-related species at the cathode–separator interface. Energy Environ. Sci..

[3] Yao W., Liao K., Lai T., Sul H., Manthiram A. (2024). Rechargeable metal-sulfur batteries: Key materials to mechanisms. Chem. Rev..

[4] Sun J., Wang T., Gao Y., Pan Z., Hu R., Wang J. (2022). Will lithium-sulfur batteries be the next beyond-lithium ion batteries and even much better?. InfoMat.

[5] Manthiram A., Fu Y.Z., Chung S.H., Zu C.X., Su Y.S. (2014). Rechargeable lithium-sulfur batteries. Chem. Rev..

[6] Trento C. Lithium-Sulfur Batteries vs. Lithium-Ion Batteries: A Comparative Analysis. https://www.samaterials.com/lithium-sulfur-batteries-lithium-ion-batteries.html.

[7] Bruce P.G., Freunberger S.A., Hardwick L.J., Tarascon J.-M. (2012). Li–O_2_ and Li–S batteries with high energy storage. Nat. Mater..

[8] Lin D., Liu Y., Cui Y. (2017). Reviving the lithium metal anode for high-energy batteries. Nat. Nanotechnol..

[9] Wickerts S., Arvidsson R., Nordelöf A., Svanström M., Johansson P. (2023). Prospective life cycle assessment of lithium-sulfur batteries for stationary energy storage. ACS Sustain. Chem. Eng..

[10] Yan C., Zhang X.-Q., Huang J.-Q., Liu Q., Zhang Q. (2019). Lithium-anode protection in lithium–sulfur batteries. Trends Chem..

[11] Hagen M., Hanselmann D., Ahlbrecht K., Maça R., Gerber D., Tübke J. (2015). Lithium–sulfur cells: The gap between the state-of-the-art and the requirements for high energy battery cells. Adv. Energy Mater..

[12] Chen Z.-X., Zhao M., Hou L.-P., Zhang X.-Q., Li B.-Q., Huang J.-Q. (2022). Toward practical high-energy-density lithium–sulfur pouch cells: A review. Adv. Mater..

[13] Gao Y., Guo Q., Zhang Q., Cui Y., Zheng Z. (2021). Fibrous materials for flexible Li–S battery. Adv. Energy Mater..

[14] Meyerson M.L., Rosenberg S.G., Small L.J. (2022). A mediated Li–S flow battery for grid-scale energy storage. ACS Appl. Energy Mater..

[15] Ni W., Shi L.-Y., Gupta R.K., Nguyen T.A., Song H., Yasin G. (2022). 14—Graphene–sulfur nanocomposites as cathode materials and separators for lithium–sulfur batteries. Lithium-Sulfur Batteries.

[16] Wang X., Tan Y., Shen G., Zhang S. (2020). Recent progress in fluorinated electrolytes for improving the performance of Li–S batteries. J. Energy Chem..

[17] Zhao M., Li B.-Q., Peng H.-J., Yuan H., Wei J.-Y., Huang J.-Q. (2020). Lithium–Sulfur batteries under lean electrolyte conditions: Challenges and opportunities. Angew. Chem. Int. Ed..

[18] Li J., Gao L., Pan F., Gong C., Sun L., Gao H., Zhang J., Zhao Y., Wang G., Liu H. (2023). Engineering strategies for suppressing the shuttle effect in lithium–sulfur batteries. Nano-Micro Lett..

[19] Yang Y., Zheng G., Cui Y. (2013). Nanostructured sulfur cathodes. Chem. Soc. Rev..

[20] Cha E., Patel M., Bhoyate S., Prasad V., Choi W. (2020). Nanoengineering to achieve high efficiency practical lithium–sulfur batteries. Nanoscale Horiz..

[21] Ni W., Cheng J., Li X., Guan Q., Qu G., Wang Z., Wang B. (2016). Multiscale sulfur particles confined in honeycomb-like graphene with the assistance of bio-based adhesive for ultrathin and robust free-standing electrode of Li–S batteries with improved performance. RSC Adv..

[22] Yang D., Ni W., Cheng J., Wang Z., Wang T., Guan Q., Zhang Y., Wu H., Li X., Wang B. (2017). Flexible three-dimensional electrodes of hollow carbon bead strings as graded sulfur reservoirs and the synergistic mechanism for lithium–sulfur batteries. Appl. Surf. Sci..

[23] Ji Z., Feng L., Zhu Z., Fu X., Yang W., Wang Y. (2023). Polymeric interface engineering in lithium-sulfur batteries. Chem. Eng. J..

[24] Zhong M.E., Guan J., Sun J., Shu X., Ding H., Chen L., Zhou N., Xiao Z. (2021). A cost- and energy density-competitive lithium-sulfur battery. Energy Storage Mater..

[25] Zhang J., Huang H., Bae J., Chung S.-H., Zhang W., Manthiram A., Yu G. (2018). Nanostructured host materials for trapping sulfur in rechargeable Li–S batteries: Structure design and interfacial chemistry. Small Methods.

[26] Wang Y., Huang X., Zhang S., Hou Y. (2018). Sulfur hosts against the shuttle effect. Small Methods.

[27] Ji X., Lee K.T., Nazar L.F. (2009). A highly ordered nanostructured carbon–sulphur cathode for lithium–sulphur batteries. Nat. Mater..

[28] Fang R., Chen K., Yin L., Sun Z., Li F., Cheng H.-M. (2019). The regulating role of carbon nanotubes and graphene in lithium-ion and lithium–sulfur batteries. Adv. Mater..

[29] Ding N., Yang J., Li X., Liu Z., Zong Y. (2019). Engineering high-performance sulfur electrode from industrial conductive carbons. ACS Sustain. Chem. Eng..

[30] Song M.-K., Zhang Y., Cairns E.J. (2013). A Long-Life, High-rate lithium/sulfur cell: A multifaceted approach to enhancing cell performance. Nano Lett..

[31] Wang Z.Y., Dong Y.F., Li H.J., Zhao Z.B., Wu H.B., Hao C., Liu S.H., Qiu J.S., Lou X.W. (2014). Enhancing lithium-sulphur battery performance by strongly binding the discharge products on amino-functionalized reduced graphene oxide. Nat. Commun..

[32] Luo C., Hu E., Gaskell K.J., Fan X., Gao T., Cui C., Ghose S., Yang X.-Q., Wang C. (2020). A chemically stabilized sulfur cathode for lean electrolyte lithium sulfur batteries. Proc. Natl. Acad. Sci. USA.

[33] Li Z., Sami I., Yang J., Li J., Kumar R.V., Chhowalla M. (2023). Lithiated metallic molybdenum disulfide nanosheets for high-performance lithium–sulfur batteries. Nat. Energy.

[34] Jo S.-C., Hong J.-W., Choi I.-H., Kim M.-J., Kim B.G., Lee Y.-J., Choi H.Y., Kim D., Kim T., Baeg K.-J. (2022). Multimodal capturing of polysulfides by phosphorus-doped carbon composites for flexible high-energy-density lithium–sulfur batteries. Small.

[35] Monisha M., Permude P., Ghosh A., Kumar A., Zafar S., Mitra S., Lochab B. (2020). Halogen-free flame-retardant sulfur copolymers with stable Li–S battery performance. Energy Storage Mater..

[36] Boenke T., Kirchhoff S., Reuter F.S., Schmidt F., Weller C., Dörfler S., Schwedtmann K., Härtel P., Abendroth T., Althues H. (2023). The role of polysulfide-saturation in electrolytes for high power applications of real world Li-S pouch cells. Nano Res..

[37] Qie L., Zu C., Manthiram A. (2016). A high energy lithium-sulfur battery with ultrahigh-loading lithium polysulfide cathode and its failure mechanism. Adv. Energy Mater..

[38] Park H., Lee S., Kim H., Park H., Kim H., Kim J., Agostini M., Sun Y.-K., Hwang J.-Y. (2024). Heterostructured nickel–cobalt metal alloy and metal oxide nanoparticles as a polysulfide mediator for stable lithium–sulfur full batteries with lean electrolyte. Carbon Energy.

[39] Xin S., Gu L., Zhao N.H., Yin Y.X., Zhou L.J., Guo Y.G., Wan L.J. (2012). Smaller sulfur molecules promise better lithium-sulfur batteries. J. Am. Chem. Soc..

[40] Zhu Q., Zhao Q., An Y., Anasori B., Wang H., Xu B. (2017). Ultra-microporous carbons encapsulate small sulfur molecules for high performance lithium-sulfur battery. Nano Energy.

[41] Pai R., Singh A., Tang M.H., Kalra V. (2022). Stabilization of gamma sulfur at room temperature to enable the use of carbonate electrolyte in Li-S batteries. Commun. Chem..

[42] Chung W.J., Griebel J.J., Kim E.T., Yoon H., Simmonds A.G., Ji H.J., Dirlam P.T., Glass R.S., Wie J.J., Nguyen N.A. (2013). The use of elemental sulfur as an alternative feedstock for polymeric materials. Nat. Chem..

[43] Zhou J., Holekevi Chandrappa M.L., Tan S., Wang S., Wu C., Nguyen H., Wang C., Liu H., Yu S., Miller Q.R.S. (2024). Healable and conductive sulfur iodide for solid-state Li–S batteries. Nature.

[44] Judez X., Martinez-Ibañez M., Santiago A., Armand M., Zhang H., Li C. (2019). Quasi-solid-state electrolytes for lithium sulfur batteries: Advances and perspectives. J. Power Sources.

[45] Liu Y., Meng X., Wang Z., Qiu J. (2022). Development of quasi-solid-state anode-free high-energy lithium sulfide-based batteries. Nat. Commun..

[46] Ding B., Wang J., Fan Z., Chen S., Lin Q., Lu X., Dou H., Kumar Nanjundan A., Yushin G., Zhang X. (2020). Solid-state lithium–sulfur batteries: Advances, challenges and perspectives. Mater. Today.

[47] Zhang S., Ueno K., Dokko K., Watanabe M. (2015). Recent advances in electrolytes for lithium–sulfur batteries. Adv. Energy Mater..

[48] Zheng G., Yang Y., Cha J.J., Hong S.S., Cui Y. (2011). Hollow carbon nanofiber-encapsulated sulfur cathodes for high specific capacity rechargeable lithium batteries. Nano Lett..

[49] Adams B.D., Carino E.V., Connell J.G., Han K.S., Cao R., Chen J., Zheng J., Li Q., Mueller K.T., Henderson W.A. (2017). Long term stability of Li-S batteries using high concentration lithium nitrate electrolytes. Nano Energy.

[50] Lei D., Shi K., Ye H., Wan Z., Wang Y., Shen L., Li B., Yang Q.-H., Kang F., He Y.-B. (2018). Progress and perspective of solid-state lithium–sulfur batteries. Adv. Funct. Mater..

[51] Pang Q., Shyamsunder A., Narayanan B., Kwok C.Y., Curtiss L.A., Nazar L.F. (2018). Tuning the electrolyte network structure to invoke quasi-solid state sulfur conversion and suppress lithium dendrite formation in Li–S batteries. Nat. Energy.

[52] Cheng L., Curtiss L.A., Zavadil K.R., Gewirth A.A., Shao Y., Gallagher K.G. (2016). Sparingly solvating electrolytes for high energy density lithium–sulfur batteries. ACS Energy Lett..

[53] Gao X., Yu Z., Wang J., Zheng X., Ye Y., Gong H., Xiao X., Yang Y., Chen Y., Bone S.E. (2023). Electrolytes with moderate lithium polysulfide solubility for high-performance long-calendar-life lithium–sulfur batteries. Proc. Natl. Acad. Sci. USA.

[54] Cheng X.-B., Huang J.-Q., Peng H.-J., Nie J.-Q., Liu X.-Y., Zhang Q., Wei F. (2014). Polysulfide shuttle control: Towards a lithium-sulfur battery with superior capacity performance up to 1000 cycles by matching the sulfur/electrolyte loading. J. Power Sources.

[55] Lee C.-W., Pang Q., Ha S., Cheng L., Han S.-D., Zavadil K.R., Gallagher K.G., Nazar L.F., Balasubramanian M. (2017). Directing the lithium–sulfur reaction pathway via sparingly solvating electrolytes for high energy density batteries. ACS Cent. Sci..

[56] Chen Z.-X., Cheng Q., Li X.-Y., Li Z., Song Y.-W., Sun F., Zhao M., Zhang X.-Q., Li B.-Q., Huang J.-Q. (2023). Cathode kinetics evaluation in lean-electrolyte lithium–sulfur batteries. J. Am. Chem. Soc..

[57] Baek M., Shin H., Char K., Choi J.W. (2020). New high donor electrolyte for lithium–sulfur batteries. Adv. Mater..

[58] Zhang X., Wang A., Liu X., Luo J. (2019). Dendrites in lithium metal anodes: Suppression, regulation, and elimination. Acc. Chem. Res..

[59] Lee H.G., Kim S.Y., Lee J.S. (2022). Dynamic observation of dendrite growth on lithium metal anode during battery charging/discharging cycles. npj Comput. Mater..

[60] Lee B., Paek E., Mitlin D., Lee S.W. (2019). Sodium metal anodes: Emerging solutions to dendrite growth. Chem. Rev..

[61] Zheng J., Ji G., Fan X., Chen J., Li Q., Wang H., Yang Y., DeMella K.C., Raghavan S.R., Wang C. (2019). High-fluorinated electrolytes for Li–S batteries. Adv. Energy Mater..

[62] Zheng J., Fan X., Ji G., Wang H., Hou S., DeMella K.C., Raghavan S.R., Wang J., Xu K., Wang C. (2018). Manipulating electrolyte and solid electrolyte interphase to enable safe and efficient Li-S batteries. Nano Energy.

[63] Yang Y., Wang H., Zhu C., Ma J. (2023). Armor-like inorganic-rich cathode electrolyte interphase enabled by the pentafluorophenylboronic acid additive for high-voltage Li||NCM622 batteries. Angew. Chem. Int. Ed..

[64] Guo W., Zhang W., Si Y., Wang D., Fu Y., Manthiram A. (2021). Artificial dual solid-electrolyte interfaces based on in situ organothiol transformation in lithium sulfur battery. Nat. Commun..

[65] Jin C., Huang Y., Li L., Wei G., Li H., Shang Q., Ju Z., Lu G., Zheng J., Sheng O. (2023). A corrosion inhibiting layer to tackle the irreversible lithium loss in lithium metal batteries. Nat. Commun..

[66] Frischmann P.D., Hwa Y., Cairns E.J., Helms B.A. (2016). Redox-active supramolecular polymer binders for lithium–sulfur batteries that adapt their transport properties in operando. Chem. Mater..

[67] Huang Y., Shaibani M., Gamot T.D., Wang M., Jovanović P., Dilusha Cooray M.C., Mirshekarloo M.S., Mulder R.J., Medhekar N.V., Hill M.R. (2021). A saccharide-based binder for efficient polysulfide regulations in Li-S batteries. Nat. Commun..

[68] Zhou G., Liu K., Fan Y., Yuan M., Liu B., Liu W., Shi F., Liu Y., Chen W., Lopez J. (2018). An aqueous inorganic polymer binder for high performance lithium–sulfur batteries with flame-retardant properties. ACS Cent. Sci..

[69] Senthil C., Kim S.-S., Jung H.Y. (2022). Flame retardant high-power Li-S flexible batteries enabled by bio-macromolecular binder integrating conformal fractions. Nat. Commun..

[70] Shaibani M., Mirshekarloo M.S., Singh R., Easton C.D., Cooray M.C.D., Eshraghi N., Abendroth T., Dörfler S., Althues H., Kaskel S. (2020). Expansion-tolerant architectures for stable cycling of ultrahigh-loading sulfur cathodes in lithium-sulfur batteries. Sci. Adv..

[71] Li Z., Jiao S., Yu D., Zhang Q., Liu K., Han J., Guo Z., Liu J., Wang L. (2021). Cationic-polymer-functionalized separator as a high-efficiency polysulfide shuttle barrier for long-life Li–S battery. ACS Appl. Energy Mater..

[72] Fan Y., Yang Z., Hua W., Liu D., Tao T., Rahman M.M., Lei W., Huang S., Chen Y. (2017). Functionalized boron nitride nanosheets/graphene interlayer for fast and long-life lithium–sulfur batteries. Adv. Energy Mater..

[73] Li B.-Q., Kong L., Zhao C.-X., Jin Q., Chen X., Peng H.-J., Qin J.-L., Chen J.-X., Yuan H., Zhang Q. (2019). Expediting redox kinetics of sulfur species by atomic-scale electrocatalysts in lithium–sulfur batteries. InfoMat.

[74] Pei H., Yang C., Wang P., Lin J., Yin L., Zhou X., Xie X., Ye Y. (2022). Efficient thermal management of lithium-sulfur batteries by highly thermally conductive LBL-assembled composite separators. Electrochim. Acta.

[75] Wang M., Emre A.E., Kim J.-Y., Huang Y., Liu L., Cecen V., Huang Y., Kotov N.A. (2022). Multifactorial engineering of biomimetic membranes for batteries with multiple high-performance parameters. Nat. Commun..

[76] Zhao C., Xu G.-L., Yu Z., Zhang L., Hwang I., Mo Y.-X., Ren Y., Cheng L., Sun C.-J., Ren Y. (2021). A high-energy and long-cycling lithium–sulfur pouch cell via a macroporous catalytic cathode with double-end binding sites. Nat. Nanotechnol..

[77] Li G., Lei W., Luo D., Deng Y.P., Wang D., Chen Z. (2018). 3D porous carbon sheets with multidirectional ion pathways for fast and durable lithium–sulfur batteries. Adv. Energy Mater..

[78] Gu X., Lai C. (2019). One dimensional nanostructures contribute better Li–S and Li–Se batteries: Progress, challenges and perspectives. Energy Storage Mater..

[79] Zheng M., Chi Y., Hu Q., Tang H., Jiang X., Zhang L., Zhang S., Pang H., Xu Q. (2019). Carbon nanotube-based materials for lithium–sulfur batteries. J. Mater. Chem. A.

[80] Shao Q., Wu Z.-S., Chen J. (2019). Two-dimensional materials for advanced Li-S batteries. Energy Storage Mater..

[81] Liu Y., Barnscheidt Y., Peng M., Bettels F., Li T., He T., Ding F., Zhang L. (2021). A biomass-based integral approach enables Li-S full pouch cells with exceptional power density and energy density. Adv. Sci..

[82] Li Y., Wang W., Zhang B., Fu L., Wan M., Li G., Cai Z., Tu S., Duan X., Seh Z.W. (2021). Manipulating redox kinetics of sulfur species using Mott–Schottky electrocatalysts for advanced lithium–sulfur batteries. Nano Lett..

[83] Li H., Meng R., Ye C., Tadich A., Hua W., Gu Q., Johannessen B., Chen X., Davey K., Qiao S.-Z. (2024). Developing high-power Li||S batteries via transition metal/carbon nanocomposite electrocatalyst engineering. Nat. Nanotechnol..

[84] Liu Y., Ma S., Liu L., Koch J., Rosebrock M., Li T., Bettels F., He T., Pfnür H., Bigall N.C. (2020). Nitrogen doping improves the immobilization and catalytic effects of Co_9_S_8_ in Li-S batteries. Adv. Funct. Mater..

[85] Tian Y., Yang M., Wang C. (2022). Highly efficient flexible Li–S full batteries with hollow Ru–RuO_2−*x*_ nanofibers as robust polysulfide anchoring-catalysts and lithium dendrite inhibitors. J. Mater. Chem. A.

[86] Deng S., Guo T., Heier J., Zhang C. (2023). Unraveling polysulfide’s adsorption and electrocatalytic conversion on metal oxides for li-s batteries. Adv. Sci..

[87] Zhou T., Liang J., Ye S., Zhang Q., Liu J. (2023). Fundamental, application and opportunities of single atom catalysts for Li-S batteries. Energy Storage Mater..

[88] Kwok C.Y., Xu S., Kochetkov I., Zhou L., Nazar L.F. (2023). High-performance all-solid-state Li_2_S batteries using an interfacial redox mediator. Energy Environ. Sci..

[89] Chung S.-H., Manthiram A. (2019). A Li_2_S-TiS_2_-electrolyte composite for stable Li_2_S-based lithium–sulfur batteries. Adv. Energy Mater..

[90] Liu D.D., Chen C., Xiong X.H. (2021). Research progress on artificial protective films for lithium metal anodes. Acta Phys.-Chim. Sin..

[91] Zhang X., Yang Y., Zhou Z. (2020). Towards practical lithium-metal anodes. Chem. Soc. Rev..

[92] Ren Y.X., Zeng L., Jiang H.R., Ruan W.Q., Chen Q., Zhao T.S. (2019). Rational design of spontaneous reactions for protecting porous lithium electrodes in lithium–sulfur batteries. Nat. Commun..

[93] Chen W., Hu Y., Liu Y., Wang S., Hu A., Lei T., Li Y., Li P., Chen D., Xia L. (2024). Ultralong cycling and safe lithium–sulfur pouch cells for sustainable energy storage. Adv. Mater..

[94] Eroglu D., Zavadil K.R., Gallagher K.G. (2015). Critical link between materials chemistry and cell-level design for high energy density and low cost lithium-sulfur transportation battery. J. Electrochem. Soc..

[95] Wolff D., Canals Casals L., Benveniste G., Corchero C., Trilla L. (2019). The effects of lithium sulfur battery ageing on second-life possibilities and environmental life cycle assessment studies. Energies.

[96] Bhargav A., He J., Gupta A., Manthiram A. (2020). Lithium-sulfur batteries: Attaining the critical metrics. Joule.

[97] Chung S.-H., Chang C.-H., Manthiram A. (2018). Progress on the critical parameters for lithium–sulfur batteries to be practically viable. Adv. Funct. Mater..

[98] Lee B.-J., Zhao C., Yu J.-H., Kang T.-H., Park H.-Y., Kang J., Jung Y., Liu X., Li T., Xu W. (2022). Development of high-energy non-aqueous lithium-sulfur batteries via redox-active interlayer strategy. Nat. Commun..

[99] Xu J., Liu K., Khan M.A., Wang H., He T., Zhao H., Ye D., Tang Y., Zhang J. (2022). Sub-zero temperature electrolytes for lithium-sulfur batteries: Functional mechanisms, challenges and perspectives. Chem. Eng. J..

[100] Kulisch J., Sommer H., Brezesinski T., Janek J. (2014). Simple cathode design for Li–S batteries: Cell performance and mechanistic insights by in operando X-ray diffraction. Phys. Chem. Chem. Phys..

[101] Fu Y., Singh R.K., Feng S., Liu J., Xiao J., Bao J., Xu Z., Lu D. (2023). Understanding of low-porosity sulfur electrode for high-energy lithium–sulfur batteries. Adv. Energy Mater..

[102] Enabling Fast Charging Batteries with 3D Lithium Metal Architectures and Sulfurized Carbon Cathodes. https://arpa-e.energy.gov/technologies/projects/enabling-fast-charging-batteries-3d-lithium-metal-architectures-and.

[103] Fotouhi A., Auger D.J., Propp K., Longo S., Wild M. (2016). A review on electric vehicle battery modelling: From lithium-ion toward lithium–sulphur. Renew. Sustain. Energy Rev..

[104] Propp K., Marinescu M., Auger D.J., O’Neill L., Fotouhi A., Somasundaram K., Offer G.J., Minton G., Longo S., Wild M. (2016). Multi-temperature state-dependent equivalent circuit discharge model for lithium-sulfur batteries. J. Power Sources.

[105] Zeng Q., Zou Z., Chen J., Jiang Y., Zeng L., Li C. (2021). Closed-loop modeling to evaluate the performance of a scaled-up lithium–sulfur battery in electric vehicle applications. Appl. Sci..

[106] Fotouhi A., Auger D.J., Propp K., Longo S. (2017). Electric vehicle battery parameter identification and SOC observability analysis: NiMH and Li-S case studies. IET Power Electron..

[107] Stroe D.I., Knap V., Swierczynski M., Schaltz E. (2019). Electrochemical impedance spectroscopy-based electric circuit modeling of lithium–sulfur batteries during a discharging state. IEEE Trans. Ind. Appl..

[108] Fotouhi A., Auger D.J., Propp K., Longo S. (2018). Lithium–sulfur battery state-of-charge observability analysis and estimation. IEEE Trans. Power Electron..

[109] Ye Y., Zhang J., Pilla S., Rao A.M., Xu B. (2023). Application of a new type of lithium-sulfur battery and reinforcement learning in plug-in hybrid electric vehicle energy management. J. Energy Storage.

[110] Li X., Lushington A., Sun Q., Xiao W., Liu J., Wang B., Ye Y., Nie K., Hu Y., Xiao Q. (2016). Safe and durable high-temperature lithium–sulfur batteries via molecular layer deposited coating. Nano Lett..

[111] Xu Y., Zhu Y., Nie T., Zhu A., Xu J., Cao Y., Hu S., Zhang X., Niu D. (2024). Interwoven N-doped bimetallic/carbon nanosheets as an efficient electrocatalyst for wide-temperature lithium-sulfur batteries. Chem. Eng. J..

[112] Deng Y., Li J., Li T., Gao X., Yuan C. (2017). Life cycle assessment of lithium sulfur battery for electric vehicles. J. Power Sources.

[113] Benveniste G., Sánchez A., Rallo H., Corchero C., Amante B. (2022). Comparative life cycle assessment of Li-Sulphur and Li-ion batteries for electric vehicles. Resour. Conserv. Recycl. Adv..

[114] Accardo A., Garofalo A., Dotelli G., Spessa E. (2024). Prospective LCA of Next-Generation Cells for Electric Vehicle Applications. IEEE Access.

[115] Zhang H., Xue B., Li S., Yu Y., Li X., Chang Z., Wu H., Hu Y., Huang K., Liu L. (2023). Life cycle environmental impact assessment for battery-powered electric vehicles at the global and regional levels. Sci. Rep..

[116] Li G., Chen Z., Lu J. (2018). Lithium-Sulfur Batteries for Commercial Applications. Chem.

[117] Benveniste G., Rallo H., Canals Casals L., Merino A., Amante B. (2018). Comparison of the state of Lithium-Sulphur and lithium-ion batteries applied to electromobility. J. Environ. Manag..

[118] Iclodean C., Varga B., Burnete N., Cimerdean D., Jurchiş B. (2017). Comparison of different battery types for electric vehicles. IOP Conf. Ser. Mater. Sci. Eng..

[119] Siczek K., Siczek K., Piersa P., Adrian Ł., Szufa S., Obraniak A., Kubiak P., Zakrzewicz W., Bogusławski G. (2020). The comparative study on the Li-S and Li-ion batteries cooperating with the photovoltaic array. Energies.

[120] Amos J. Solar Plane Makes Record Flight. http://news.bbc.co.uk/1/hi/sci/tech/7577493.stm.

[121] Sino Power INFORMATION BRIEF: Transitioning from Lithium-Sulfur to Licerion Technology. https://sionpower.com/files/IB_Li-S2Licerion.pdf.

[122] Kane M. OXIS to Offer 450 Wh/kg Quasi Solid-State Li-S Cells in Fall 2021. https://insideevs.com/news/502505/oxis-450-whkg-solidstate-cells/.

[123] Lithium-Sulfur Batteries One Step Closer to Commercialisation in the UK: A Faraday Success Story. https://www.innovationnewsnetwork.com/lithium-sulfur-batteries-one-step-closer-to-commercialisation-in-the-uk/41609/.

[124] OXIS Energy to Supply Solid-State Li-S Batteries for Trials and Testing by This Fall; 600 Wh/kg and 900 Wh/L by 2026. https://www.greencarcongress.com/2021/04/20210421-oxis.html.

[125] Alcock C. Patents for Sale as Battery Specialist Oxis Collapses. https://www.futureflight.aero/news-article/2021-05-27/patents-sale-battery-specialist-oxis-collapses.

[126] Avalos G. Lyten Launches San Jose Pilot Production for Cutting-Edge Batteries. https://www.mercurynews.com/2023/06/14/lyten-san-jose-battery-economy-tech-jobs-real-estate-green-car-energy/.

[127] Li-S Energy Achieves 45% Increase in Volumetric Energy Density with New Semi-Solid State Lithium Sulfur Battery. https://www.businesswire.com/news/home/20230529005039/en/.

[128] Li-S Energy Achieves 45% Increase in Volumetric Energy Density with New 20-Layer Semi-Solid State Lithium Sulfur Battery. https://chargedevs.com/newswire/li-s-energy-claims-to-boost-energy-density-by-45-with-new-20-layer-lithium-sulfur-battery/.

[129] Zeta Energy Corporation. https://zetaenergy.com/.

[130] Davies D.M., Verde M.G., Mnyshenko O., Chen Y.R., Rajeev R., Meng Y.S., Elliott G. (2019). Combined economic and technological evaluation of battery energy storage for grid applications. Nat. Energy.

[131] Comello S., Reichelstein S. (2019). The emergence of cost effective battery storage. Nat. Commun..

[132] Chen T., Jin Y., Lv H., Yang A., Liu M., Chen B., Xie Y., Chen Q. (2020). Applications of lithium-ion batteries in grid-scale energy storage systems. Trans. Tianjin Univ..

[133] Zhang S., Guo W., Yang F., Zheng P., Qiao R., Li Z. (2019). Recent progress in polysulfide redox-flow batteries. Batter. Supercaps.

[134] Pan H., Wei X., Henderson W.A., Shao Y., Chen J., Bhattacharya P., Xiao J., Liu J. (2015). On the way toward understanding solution chemistry of lithium polysulfides for high energy Li–S redox flow batteries. Adv. Energy Mater..

[135] Khan I.A., Alzahrani A.S., Ali S., Mansha M., Tahir M.N., Khan M., Qayyum H.A., Khan S.A. (2024). Development of membranes and separators to inhibit cross-shuttling of sulfur in polysulfide-based redox flow batteries: A review. Chem. Rec..

[136] Dörfler S., Walus S., Locke J., Fotouhi A., Auger D.J., Shateri N., Abendroth T., Härtel P., Althues H., Kaskel S. (2021). Recent progress and emerging application areas for lithium–sulfur battery technology. Energy Technol..

